# The genus *Melanconis* (Diaporthales)

**DOI:** 10.3897/mycokeys.63.49054

**Published:** 2020-03-02

**Authors:** Walter M. Jaklitsch, Hermann Voglmayr

**Affiliations:** 1 Institute of Forest Entomology, Forest Pathology and Forest Protection, Department of Forest and Soil Sciences, BOKU-University of Natural Resources and Life Sciences, Franz Schwackhöfer Haus, Peter-Jordan-Straße 82/I, 1190 Vienna, Austria University of Natural Resources and Life Sciences Vienna Austria; 2 Division of Systematic and Evolutionary Botany, Department of Botany and Biodiversity Research, University of Vienna, Rennweg 14, 1030 Wien, Austria University of Vienna Vienna Austria

**Keywords:** *
Juglanconis
*, *
Melanconiella
*, *
Melanconium
*, multigene phylogeny, pyrenomycetes, systematics, 1 new combination, 2 new species

## Abstract

The genus *Melanconis* (Melanconidaceae, Diaporthales) in the strict sense is here re-evaluated regarding phylogenetic structure, taxonomy, distribution and ecology. Using a matrix of sequences from ITS, LSU, *ms204*, *rpb2*, *tef1* and *tub2*, eight species are recognised and their phylogenetic positions are determined. Based on phylogenetic, morphological and geographical differentiation, *Melanconis marginalis* is subdivided into four subspecies. *Melanconis italica* is reduced to a subspecies of *Melanconis marginalis*. The two species *Melanconis larissae* from *Betula* sp. and *M. pacifica* from *Alnus rubra* are described as new. *Melanconis alni* and *M. stilbostoma* are lectotypified and *M. alni*, *M. marginalis* and *M. stilbostoma* are epitypified. All GenBank sequences deposited as *Melanconis alni* are shown to actually represent *M. marginalis* and those as *M. marginalis* belong to the newly described *M. pacifica*. Currently, *Alnus* and *Betula* are the sole host genera of *Melanconis*. All species and subspecies are (re-)described and illustrated. In addition, the neotypification of *Melanconium pterocaryae* is here validated.

## Introduction

*Melanconis*, the type genus of the family Melanconidaceae (Diaporthales), was originally described by Tulasne (1856) with *M. stilbostoma* as its generic type, but without a generic diagnosis. His inclusion of species like *M. spodiaea* made the genus heterogeneous from the beginning. Since then, many species names have been erected in the genus. In his generic revision, Wehmeyer (1941) treated the genus in a very wide sense, organising the species in subgenera and sections, which themselves were heterogeneous, containing species of genera like *Chapeckia*, Coryneum (Pseudovalsa), *Macrodiaporthe*, *Massariovalsa*, *Melanconiella* or *Pseudovalsella*. Barr (1978) accepted Melanconis roughly in the sense of Wehmeyer´s subgenus Eumelanconis, which included *Melanconiella*. In this sense, the genus *Melanconis* was one of many genera of the large family Melanconidaceae and was defined by a distinct ectostromatic disc, a more or less well-developed entostroma, two-celled hyaline or brown ascospores with or without appendages, in combination with melanconium- or discosporium-like asexual morphs (Barr 1978). The first phylogenetic analyses of the Diaporthales (Castlebury et al. 2002; see also Jaklitsch et al. 2016, Senanayake et al. 2018), however, suggested that Melanconidaceae should be confined to its type genus *Melanconis* with a restricted number of species. This phylogenetic generic concept corresponds, apart from a few exceptions, with Wehmeyer’s (1941) section Stilbostomae of his subgenus Eumelanconis. Subsequently, many names have been combined in other genera in various families following morphological and/or phylogenetic analyses (Barr 1978; Jaklitsch and Voglmayr 2004; Voglmayr and Jaklitsch 2008; De Silva et al. 2009). *Melanconiella* was extensively studied by Voglmayr et al. (2012), who determined that species of *Melanconis* cause more conspicuous bumps in the host bark than those of *Melanconiella* and form light-coloured, white or yellowish ectostromatic discs. Wehmeyer (1941) had used this trait to distinguish his section Stilbostomae from his Chrysostromae, which are characterised by dark coloured discs. Although light coloured discs are not uncommon in *Melanconiella*, Wehmeyer’s (1941) section Chrysostromae of his subgenus Eumelanconis basically matches the phylogenetically conceived genus *Melanconiella*, except for a few species, which belong elsewhere. For some of these species, the new genus *Juglanconis* was established in the new family Juglanconidaceae (Voglmayr et al. 2017, 2019). Two other species were segregated from *Melanconis* to *Alnecium* and *Phaeodiaporthe* by Voglmayr and Jaklitsch (2014). Voglmayr et al. (2012) found an unexpectedly high species diversity in *Melanconiella*, particularly on *Carpinus* spp. and showed that its species either have a melanconium- or a discosporina-like asexual morph, but never both morph types. They gave also information of taxonomic placement of other *Melanconis* spp. Here we treat the residual species of *Melanconis* in the strict sense.

## Materials and methods

### Sample sources

All isolates included in this study originated from ascospores or conidia of freshly collected specimens derived from recently dead branches or twigs. Details of the strains including NCBI GenBank accession numbers of gene sequences used to compute the phylogenetic trees are listed in Table 1. Strain acronyms, other than those of official culture collections, are used here primarily as strain identifiers throughout the work. Representative isolates have been deposited at the Westerdijk Fungal Biodiversity Centre (CBS-KNAW), Utrecht, The Netherlands. Details of the specimens, used for morphological investigations, are listed in the Taxonomy section under the respective descriptions. Herbarium acronyms are according to Thiers (2019). Freshly collected specimens have been deposited in the Fungarium of the Department of Botany and Biodiversity Research, University of Vienna (WU) and in the Fungarium of the Natural History Museum of Vienna (W).

**Table 1.** Isolates and accession numbers of sequences used in the phylogenetic analyses.

Taxon	Strain^1^	Origin	Host	GenBank accession no.^2^					
				ITS	LSU	*ms204*	*rpb2*	*tef1*	*tub2*
*Juglanconis appendiculata*	MC	Greece	*Juglans regia*	KY427141	KY427141	KY427159	KY427191	KY427210	KY427227
*Juglanconis japonica*	MAFF 410079 = ME20*	Japan	*Pterocarya rhoifolia*	KY427155	KY427155	KY427172	KY427205	KY427224	KY427240
*Juglanconis juglandina*	CBS 133343 = ME22	Austria	*Juglans regia*	KY427149	KY427149	KY427166	KY427199	KY427218	KY427234
*Juglanconis oblonga*	CBS 133344 = ME14	USA	*Juglans cinerea*	KY427151	KY427151	KY427168	KY427201	KY427220	KY427236
*Juglanconis pterocaryae*	CBS 144326 = D272*	Austria	*Pterocarya fraxinifolia*	MK229175	MK229175	MK238314	MK238324	MK238332	MK238338
*Melanconis alni*	CBS 131693 = MAMI	Austria	*Alnus glutinosa*	**MN784962**	**MN784962**	**MN780721**	**MN780745**	**MN780774**	**MN780803**
	CBS 131695 = MAW* (from ascospores)	Austria	*Alnus glutinosa*	**MN784963**	**MN784963**	**MN780722**	**MN780746**	**MN780775**	**MN780804**
	MEW*(from conidia)	Austria	*Alnus glutinosa*	**MN784964**	**MN784964**	**MN780723**	**MN780747**	**MN780776**	**MN780805**
	MAIV	France	*Alnus incana*	**MN784965**	**MN784965**	**MN780724**	**MN780748**	**MN780777**	**MN780806**
	D156	Poland	*Alnus glutinosa*	**MN784966**	**MN784966**	**MN780725**	**MN780749**	**MN780778**	**MN780807**
*Melanconis betulae*	CFCC 50471*	China	*Betula albosinensis*	KT732952	KT732971	–	KT732984	KT733001	KT733022
	CFCC 50472	China	*Betula albosinensis*	KT732953	KT732972	–	KT732985	KT733002	KT733023
	CFCC 50473	China	*Betula albosinensis*	KT732954	KT732973	–	KT732986	KT733003	KT733024
*Melanconis groenlandica*	CBS 116450 = UPSC 3407*	Denmark (Greenland)	*Betula nana*	KU878552	KU878553	–	–	KU878554	KU878555
	MAFF 410219 = M4-2 = ME1	Japan	*Betula maximowicziana*	**MN784967**	**MN784967**	**MN780726**	**MN780750**	**MN780779**	**MN780808**
	CBS 133341 = LCM191.01 = ME10	USA	*Betula papyrifera*	**MN784968**	**MN784968**	**MN780727**	**MN780751**	**MN780780**	**MN780809**
	CBS 133339 = LCM 02.02 = ME13	USA	*Betula* sp.	**MN784969**	**MN784969**	**MN780728**	**MN780752**	**MN780781**	**MN780810**
	CBS 133340 = LCM 185.01	USA	*Betula papyrifera*	**MN784970**	**MN784970**	**MN780729**	**MN780753**	**MN780782**	**MN780811**
*Melanconis itoana*	MAFF 410080 = LFP-M4-9 = ME8	Japan	*Betula ermanii*	**MN784971**	**MN784971**	**MN780730**	**MN780754**	**MN780783**	**MN780812**
	CFCC 50474	China	*Betula albosinensis*	KT732955	KT732974	–	KT732987	KT733004	KT733025
	CFCC 52876	China	*Betula albosinensis*	MK096324	MK096364	–	MK096409	MK096284	–
	CFCC 52877	China	*Betula albosinensis*	MK096326	MK096366	–	MK096411	MK096286	–
	CFCC 52878	China	*Betula albosinensis*	MK096327	MK096367	–	MK096412	MK096287	–
*Melanconis larissae*	CBS 123196 = AR 3886 = ME7*	USA	*Betula* sp.	**MN784972**	**MN784972**	**MN780731**	**MN780755**	**MN780784**	**MN780813**
Melanconis marginalis subsp. europaea	D157	Austria	*Alnus alnobetula*	**MN784973**	**MN784973**	–	**MN780756**	**MN780785**	–
	D158	Austria	*Alnus alnobetula*	**MN784974**	**MN784974**	**MN780732**	**MN780757**	**MN780786**	**MN780814**
	D257	Austria	*Alnus incana*	**MN784975**	**MN784975**	–	**MN780758**	**MN780787**	**MN780815**
	CBS 131692 = MAI*	Austria	*Alnus incana*	**MN784976**	**MN784976**	**MN780733**	**MN780759**	**MN780788**	**MN780816**
	CBS 131694 = MAV	Austria	*Alnus alnobetula*	**MN784977**	**MN784977**	**MN780734**	**MN780760**	**MN780789**	**MN780817**
	MAV1	Austria	*Alnus alnobetula*	**MN784978**	**MN784978**	**MN780735**	**MN780761**	**MN780790**	**MN780818**
Melanconis marginalis subsp. italica	MFLUCC 16-1199*	Italy	*Alnus cordata*	MF190151	MF190096	–	–	–	–
	MFLUCC 17-1659*	Italy	*Alnus cordata*	MF190152	MF190097	–	MF377602	–	–
Melanconis marginalis subsp. marginalis	D321 (from ascospores)*	Canada	Alnus alnobetula subsp. crispa	**MN784979**	**MN784979**	–	**MN780762**	**MN780791**	**MN780819**
	D321a (from α-conidia)*	Canada	Alnus alnobetula subsp. crispa	**MN784980**	**MN784980**	–	**MN780763**	**MN780792**	**MN780820**
	D321b (from β-conidia)*	Canada	Alnus alnobetula subsp. crispa	**MN784981**	**MN784981**	–	**MN780764**	**MN780793**	**MN780821**
	CBS 109496 = AR 3529 = ME2	Russia	Alnus alnobetula subsp. maximowiczii	**MN784982**	**MN784982**	**MN780736**	**MN780765**	**MN780794**	**MN780822**
	AR 4864 = ME5	USA	*Alnus alnobetula*	**MN784983**	**MN784983**	**MN780737**	**MN780766**	**MN780795**	**MN780823**
	CBS 133346 = AR 4865 = ME6	USA	*Alnus alnobetula*	**MN784984**	**MN784984**	**MN780738**	**MN780767**	**MN780796**	**MN780824**
	MAFF 410218 = M4-6 = ME9	Japan	Alnus alnobetula subsp. maximowiczii	**MN784985**	**MN784985**	**MN780739**	**MN780768**	**MN780797**	**MN780825**
Melanconis marginalis subsp. tirolensis	CBS 122310 = AR 3748 = ME4*	Austria	*Alnus alnobetula*	**MN784986**	**MN784986**	**MN780740**	**MN780769**	**MN780798**	**MN780826**
	D322a	Austria	*Alnus alnobetula*	**MN959458**	**MN959458**	–	**MN989415**	**MN989416**	**MN989417**
*Melanconis pacifica*	CBS 109744 = AR 3442 = AFTOL-ID 2128	Canada	*Alnus rubra*	EU199197	AF408373	–	DQ862022	DQ862038	EU219103, DQ862038
*Melanconis stilbostoma*	D143	Poland	*Betula pendula*	KY427156	KY427156	KY427173	KY427206	KY427225	KY427241
	D258	Italy	*Betula aetnensis*	**MN784987**	**MN784987**	–	**MN780770**	**MN780799**	**MN780827**
	CBS 109778 = AR 3501 = AFTOL-ID 936 = ME11*	Austria	*Betula pendula*	**MN784988**	**MN784988**	**MN780741**	**MN780771**	**MN780800**	**MN780828**
	MAFF 410225 = M3-9 = ME12	Japan	Betula platyphylla var. japonica	**MN784989**	**MN784989**	**MN780742**	**MN780772**	**MN780801**	**MN780829**
	CBS 121894 = MS	Austria	*Betula pendula*	KY427156	KY427156	**MN780743**	JQ926302	JQ926368	**MN780830**
	CBS 133338 = DMW 514.3	USA	*Betula papyrifera*	**MN784990**	**MN784990**	**MN780744**	**MN780773**	**MN780802**	**MN780831**
	CFCC 50475	China	*Betula platyphylla*	KT732956	KT732975	–	KT732988	KT733005	KT733026
	CFCC 50476	China	*Betula platyphylla*	KT732957	KT732976	–	KT732989	KT733006	KT733027
	CFCC 50477	China	*Betula platyphylla*	KT732958	KT732977	–	KT732990	KT733007	KT733028
	CFCC 50478	China	*Betula platyphylla*	KT732959	KT732978	–	KT732991	KT733008	KT733029
	CFCC 50479	China	*Betula platyphylla*	KT732960	KT732979	–	KT732992	KT733009	KT733030
	CFCC 50480	China	*Betula platyphylla*	KT732961	KT732980	–	KT732993	KT733010	KT733031
	CFCC 50481	China	*Betula platyphylla*	KT732962	KT732981	–	KT732994	KT733011	KT733032
	CFCC 50482	China	*Betula platyphylla*	KT732963	KT732982	–	KT732995	KT733012	KT733033
	CFCC 50483	China	*Betula platyphylla*	KT732964	KT732983	–	KT732996	KT733013	KT733034
	CFCC 52843	China	*Betula platyphylla*	MK096338	MK096378	–	MK096423	MK096298	–
	CFCC 52844	China	*Betula platyphylla*	MK096341	MK096381	–	MK096426	MK096301	–
	CFCC 52845	China	*Betula platyphylla*	MK096343	MK096383	–	MK096428	MK096303	–
*Melanconis stilbostoma*	CFCC 52846	China	*Betula platyphylla*	MK096347	MK096387	–	MK096432	MK096307	–
	CFCC 52847	China	*Betula platyphylla*	MK096348	MK096388	–	MK096433	MK096308	–
	CFCC 52848	China	*Betula platyphylla*	MK096349	MK096389	–	MK096434	MK096309	–
	CFCC 52849	China	*Betula platyphylla*	MK096328	MK096368	–	MK096413	MK096288	–
	CFCC 52850	China	*Betula platyphylla*	MK096329	MK096369	–	MK096414	MK096289	–
	CFCC 52851	China	*Betula platyphylla*	MK096330	MK096370	–	MK096415	MK096290	–
	CFCC 52852	China	*Betula platyphylla*	MK096331	MK096371	–	MK096416	MK096291	–
	CFCC 52853	China	*Betula platyphylla*	MK096332	MK096372	–	MK096417	MK096292	–
	CFCC 52854	China	*Betula platyphylla*	MK096333	MK096373	–	MK096418	MK096293	–
	CFCC 52855	China	*Betula platyphylla*	MK096334	MK096374	–	MK096419	MK096294	–
	CFCC 52856	China	*Betula platyphylla*	MK096335	MK096375	–	MK096420	MK096295	–
	CFCC 52857	China	*Betula platyphylla*	MK096336	MK096376	–	MK096421	MK096296	–
	CFCC 52858	China	*Betula platyphylla*	MK096337	MK096377	–	MK096422	MK096297	–
	CFCC 52859	China	*Betula platyphylla*	MK096339	MK096379	–	MK096424	MK096299	–
	CFCC 52860	China	*Betula platyphylla*	MK096340	MK096380	–	MK096425	MK096300	–
	CFCC 52861	China	*Betula platyphylla*	MK096342	MK096382	–	MK096427	MK096302	–
	CFCC 52862	China	*Betula platyphylla*	MK096344	MK096384	–	MK096429	MK096304	–
	CFCC 52863	China	*Betula platyphylla*	MK096345	MK096385	–	MK096430	MK096305	–
	CFCC 52864	China	*Betula platyphylla*	MK096346	MK096386	–	MK096431	MK096306	–
	CFCC 52865	China	*Betula platyphylla*	MK096316	MK096356	–	MK096401	MK096276	–
	CFCC 52866	China	*Betula platyphylla*	MK096317	MK096357	–	MK096402	MK096277	–
	CFCC 52867	China	*Betula platyphylla*	MK096318	MK096358	–	MK096403	MK096278	–
	CFCC 52868	China	*Betula platyphylla*	MK096319	MK096359	–	MK096404	MK096279	–
	CFCC 52869	China	*Betula platyphylla*	MK096320	MK096360	–	MK096405	MK096280	–
	CFCC 52870	China	*Betula platyphylla*	MK096321	MK096361	–	MK096406	MK096281	–
	CFCC 52871	China	*Betula platyphylla*	MK096322	MK096362	–	MK096407	MK096282	–
	CFCC 52872	China	*Betula platyphylla*	MK096323	MK096363	–	MK096408	MK096283	–
	CFCC 52873	China	*Betula platyphylla*	MK096350	MK096390	–	MK096435	MK096310	–
	CFCC 52874	China	*Betula platyphylla*	MK096351	MK096391	–	MK096436	MK096311	–
	CFCC 52875	China	*Betula platyphylla*	MK096325	MK096365	–	MK096410	MK096285	–

^1^ Ex-type strains marked by an asterisk; ^2^ Sequences in bold were generated in the present study

### Morphology

Microscopic observations were made in tap water, except where noted. Morphological analyses of microscopic characters were carried out as described by Jaklitsch (2009). Methods of microscopy included stereomicroscopy using a Nikon SMZ 1500 and Nomarski differential interference contrast (DIC), using the compound microscopes Nikon Eclipse E600 or Zeiss Axio Imager.A1 equipped with a Zeiss Axiocam 506 colour digital camera. Images and data were gathered using a Nikon Coolpix 4500 or a Nikon DS-U2 digital camera and measured by using the NIS-Elements D v. 3.0 or 3.22.15 or Zeiss ZEN Blue Edition software packages. For certain images of ascomata, the stacking software Zerene Stacker v. 1.04 (Zerene Systems LLC, Richland, WA, USA) was used. Measurements are reported as maxima and minima in parentheses and the range representing the mean plus and minus the standard deviation of the number of measurements given in parentheses.

### Culture preparation, DNA extraction, PCR and sequencing

Ascospore isolates were prepared and grown on 2% corn meal dextrose agar (CMD; CMA: Sigma, St Louis, Missouri; supplemented with 2% (w/v) D(+)-glucosemonohydrate) or 2% malt extract agar (MEA; 2% w/v malt extract, 2% w/v agar-agar; Merck, Darmstadt, Germany). Growth of liquid cultures and extraction of genomic DNA was performed as reported previously (Voglmayr and Jaklitsch 2011; Jaklitsch et al. 2012) using the DNeasy Plant Mini Kit (QIAgen GmbH, Hilden, Germany). The following loci were amplified and sequenced: a ca. 1.6 kb fragment containing the terminal part of the small subunit nuclear ribosomal DNA (nSSU rDNA), the complete internal transcribed spacer region (ITS1-5.8S-ITS2) and a ca. 900 bp fragment of the large subunit nuclear ribosomal DNA (nLSU rDNA), amplified and sequenced as a single fragment with primers V9G (De Hoog and Gerrits van den Ende 1998) and LR5 (Vilgalys and Hester 1990); a ca. 1 kb fragment of the guanine nucleotide-binding protein subunit beta (*ms204*) gene with primers MS-E1F1 and MS-E5R1 (Walker et al. 2012); a ca. 1.2 kb fragment of the RNA polymerase II subunit 2 (*rpb2*) gene with primers fRPB2-5F and fRPB2-7cR (Liu et al. 1999) or dRPB2-5f and dRPB2-7r (Voglmayr et al. 2016); and a ca. 1.3–1.5 kb fragment of the translation elongation factor 1-alpha (*tef1*) gene with primers EF1-728F (Carbone and Kohn 1999) and TEF1LLErev (Jaklitsch et al. 2005). For the β-tubulin (*tub2*) gene, either a ca. 0.45 kb fragment was amplified with primers T1 (O’Donnell and Cigelnik 1997) and BtHV2r (Voglmayr et al. 2016) or a ca. 1.6 kb fragment with primer pairs T1 and T22 (O’Donnell and Cigelnik 1997) or T1D and T22D (Voglmayr et al. 2019).

PCR products were purified using an enzymatic PCR cleanup (Werle et al. 1994), as described in Voglmayr and Jaklitsch (2008). DNA was cycle-sequenced using the ABI PRISM Big Dye Terminator Cycle Sequencing Ready Reaction Kit v. 3.1 (Applied Biosystems, Warrington, UK) and the PCR primers; in addition, primers ITS4 (White et al. 1990), LR2R-A (Voglmayr et al. 2012) and LR3 (Vilgalys and Hester 1990) were used for the SSU-ITS-LSU region, TEF1_INTF (forward, Jaklitsch 2009) and TEFD_iR1 (reverse, Jaklitsch and Voglmayr 2019) or TEF1_INT2 (reverse, Voglmayr and Jaklitsch 2017) for *tef1* and BtHVf (Voglmayr and Mehrabi 2018) and BtHV2r for the long fragment of *tub2*. Sequencing was performed on an automated DNA sequencer (3730xl Genetic Analyzer, Applied Biosystems).

### Phylogenetic analyses

The newly generated sequences were aligned with the *Melanconis* sequences of Fan et al. (2016, 2018) and a few additional GenBank sequences. Species of *Juglanconis* were selected as outgroup (Voglmayr et al. 2017, 2019); the GenBank accession numbers of the sequences, used in the phylogenetic analyses, are given in Table 1. All alignments were produced with the server version of MAFFT (www.ebi.ac.uk/Tools/mafft), checked and refined using BioEdit v. 7.2.6 (Hall 1999). For phylogenetic analyses, all sequence alignments (ITS, LSU, *ms204*, *rpb2*, *tef1* and *tub2*) were combined.

Maximum Likelihood (ML) analyses were performed with RAxML (Stamatakis 2006) as implemented in raxmlGUI 1.3 (Silvestro and Michalak 2012), using the ML + rapid bootstrap setting and the GTRGAMMA substitution model with 1000 bootstrap replicates. The matrix was partitioned for the different gene regions and substitution model parameters were calculated separately for them.

Maximum Parsimony (MP) analyses were performed with PAUP v. 4.0a166 (Swofford 2002). All molecular characters were unordered and given equal weight; analyses were performed with gaps treated as missing data; the COLLAPSE command was set to MINBRLEN. MP analysis of the combined multilocus matrix was done, using a parsimony ratchet approach. For this, a nexus file was prepared using PRAP v. 2.0b3 (Müller 2004), implementing 10000 ratchet replicates with 25% of randomly chosen positions upweighted to 2, which were then run with PAUP. MP bootstrap analyses were performed with 1000 replicates, using 5 rounds of random sequence addition and subsequent TBR branch swapping (MULTREES option in effect, steepest descent option not in effect) during each bootstrap replicate, with each replicate limited to 100000 rearrangements.

In the Results and Discussion sections, bootstrap values (BS) below 70% are considered low, between 70–90% medium and above 90% high.

## Results

### Revision of *Melanconis* sequences deposited in GenBank

Comparison of our sequences with GenBank sequences revealed that all accessions of *Melanconis alni* and *M. marginalis*, deposited in GenBank, were misidentified. All GenBank accessions of *M. alni* were shown to actually represent *M. marginalis*, while the single isolate of *M. marginalis* turned out to be a new species, described as *M. pacifica* below. These misidentifications were also confirmed by morphological re-investigation of specimens from which these sequences were generated.

### Phylogenetic analyses

**Figure 1.**
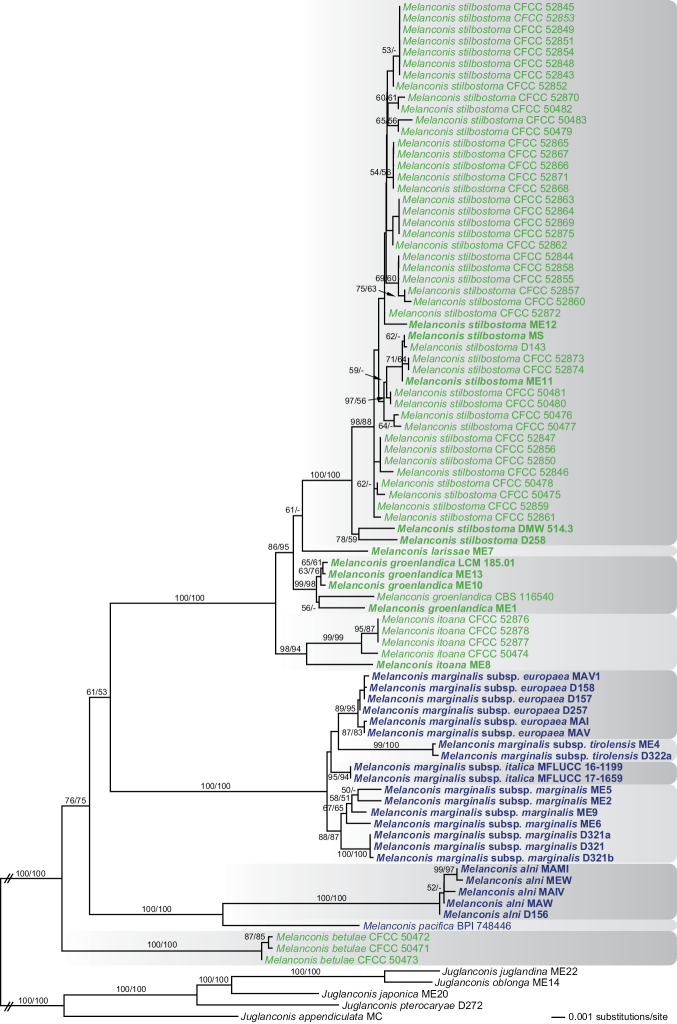
Phylogram of the best ML tree (lnL = −18240.558) revealed by RAxML from an analysis of the ITS-LSU-*ms204*-*rpb2*-*tef1*-*tub2* matrix of *Melanconis*, with 5 species of *Juglanconis* (Juglanconidaceae) selected as outgroup. ML and MP bootstrap support above 50% are given at the first and second position, respectively, above or below the branches. Strain numbers are given following the taxon names; strains formatted in bold were sequenced in the current study. *Melanconis* taxa occurring on *Alnus* are marked blue, those on *Betula* in green. The broken branches to the outgroup were scaled to 10%.

Of the 6052 characters included in the combined multilocus analyses, 925 were parsimony informative (133 from ITS-LSU, 142 from *ms204*, 214 from *rpb2*, 245 from *tef1* and 191 from *tub2*). The best ML tree (lnL = −18240.558) revealed by RAxML is shown as Fig. 1. The MP analysis revealed 3394 MP trees 1647 steps long, which were identical except for some differences within species and a polytomy at the *M. groenlandica*-*M. larissae*-*M. stilbostoma* node (not shown). Tree topology of the MP strict consensus tree was compatible with the ML tree, except for a sister group relationship of M. marginalis subsp. europaea and M. marginalis subsp. marginalis and some minor topological differences within species and subspecies (not shown).

All species of *Melanconis* received high (*M. itoana*, *M. groenlandica*) to maximum (*M. alni*, *M. betulae*, *M. marginalis*, *M. stilbostoma*) support in both analyses (Fig. 1). Sister group relationship of *M. alni* and *M. pacifica* and monophyly of the three betulicolous species *M. groenlandica*, *M. larissae* and *M. stilbostoma* received maximum support as well. Within *Melanconis marginalis*, two main subclades were evident with ML and MP BS above 85%, one containing accessions from eastern Canada, Alaska, Japan and the Russian Far East and another with accessions from Central Europe; in addition to these two main subclades, the *Melanconis marginalis* clade contained two deviating lineages, an Italian collection from ?*Alnus cordata* described as *M. italica* by Senanayake et al. (2017) and two accessions from eastern Tyrol from *Alnus alnobetula*. In light of this geographical differentiation, a substantial genetic variability within these clades (Fig. 1) and minor morphological differences, these four lineages are formally recognised on the subspecies level.

### Culture characteristics

**Figure 2.**
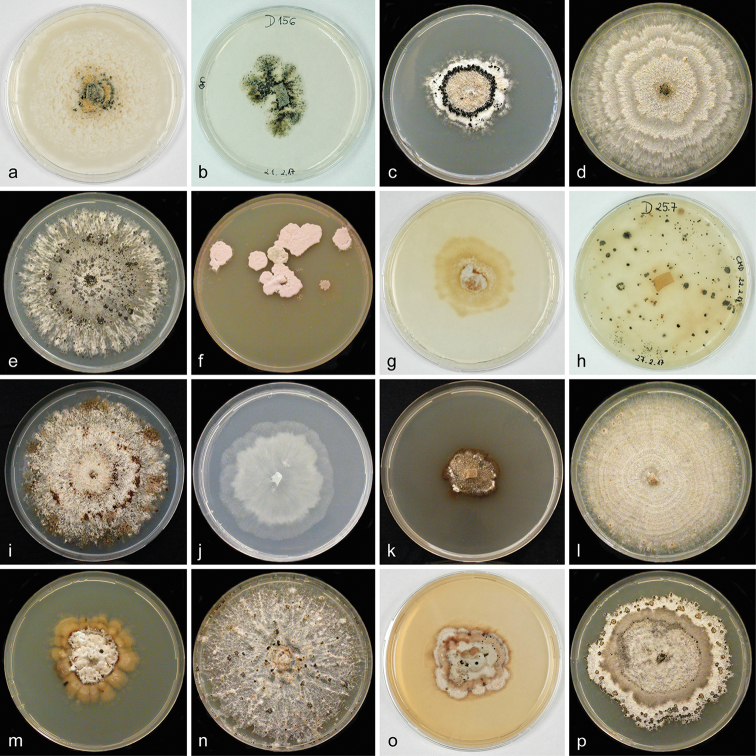
*Melanconis* cultures. **a–c***M. alni* (**a, b** D156, **c** MAW) **d***M. groenlandica* ME13 **e***M. itoana* ME8 **f***M. larissae* ME7 (after irregular rehydration) **g–i**M. marginalis subsp. europaea (**g, h** D257, **i** MAI) **j–l**M. marginalis subsp. marginalis (**j, k** D321, **l** ME5) **m**M. marginalis subsp. tirolensis ME4 **n***M. pacifica* ME3 **o, p***M. stilbostoma* (**o** D143, **p** ME11) **a, b, g, h, j, o** on CMD**c–f, i, k, l–n, p** on MEA**a, b, g, h, j** at 16 °C, **j, k** at 22 °C **c–f, i, k, l–n, p** at room temperature **a, g, j, k** after 3 weeks **b, h** after 3 **c, i** 5 **d–f, l–n, p** 3.7 **o** 2 months.

Culture images of seven studied *Melanconis* species, grown on MEA and CMD, are illustrated in Figure 2. Culture descriptions are given under the respective species.

### Taxonomy

#### Melanconis

Taxon classificationFungiDiaporthalesMelanconidaceae

Tul. & C. Tul., Select. fung. carpol. (Paris) 2: 115 (1863).

31474D83-9B28-5A4C-A9FE-277EB3A3BC31

?= Melanconium Link : Fr., Mag. Gesell. naturf. Freunde, Berlin 3(1–2): 9 (1809).

##### Type species.

*Melanconis stilbostoma* (Fr. : Fr.) Tul. & C. Tul., Select. fung. carpol. (Paris) 2: 115 (1863).

##### Notes.

Tulasne (1856) had already mentioned *Melanconis*, but did not give a generic diagnosis. Hence, the species he newly described were invalid, but became validated by reference in Tulasne and Tulasne (1863) (Paul Kirk, pers. comm.).

In contrast to *Diaporthe*, species of *Melanconis* always develop in bark, never in wood and lack stromatic zones. Pseudostromata are pulvinate to conical, circular to elliptic in outline and usually slightly project beyond the bark surface with perithecial contours remaining indistinct. Ectostromatic discs usually project distinctly from the surface of the pseudostromata and are bright, white to yellowish, to brown when old.

Nomenclaturally, the older genus *Melanconium* potentially competes with the younger genus *Melanconis*. However, as outlined in Rossman et al. (2015), the generic concept of *Melanconium* and the true identity of its generic type, *M. atrum*, are obscure and they therefore recommended to protect the well-defined *Melanconis* over *Melanconium*, which was formally adopted in the last ICN (Turland et al. 2018, Appendix III).

#### Melanconis alni

Taxon classificationFungiDiaporthalesMelanconidaceae

Tul. & C. Tul., Select. fung. carpol. (Paris) 2: 122 (1863).

3EC30B0B-3179-50CE-B3FD-C1E6BC24605E

Figures 3 , 4

≡ Melanconis alni Tul., Annls Sci. Nat., Bot., sér. 4, 5: 109 (1856). (Nom. inval., Art. 35.1).
= Rehm, Ascom. exs. 148 (1872).
?= Melanconium apiocarpum Link, in Willdenow, Sp. pl., Edn 4 6(2): 90 (1825).
?= M. sphaeroideum Link, in Willdenow, Sp. pl., Edn 4 6(2): 92 (1825).
?= Stilbospora microsperma Pers., Observ. mycol. (Lipsiae) 1: 31 (1796).

##### Diagnosis.

*Melanconis alni* is recognised by ascospores having filiform, tapering appendages and dark brown α-conidia with a pale to subhyaline median area.

##### Type material.

***Lectotype***, here designated: France, Hauts-de-Seine, Chaville, on *Alnus glutinosa*, 1 Feb 1856, Tulasne (PC 0723592; MBT390380). ***Epitype***, here designated: Austria, Oberösterreich, Raab, Wetzlbach, grid square 7648/1, on *Alnus glutinosa*, 4 Jun 2011, H. Voglmayr (WU 31883; ex-epitype cultures CBS 131695 = MAW (from ascospores), MEW (from α-conidia); MBT390381).

**Figure 3.**
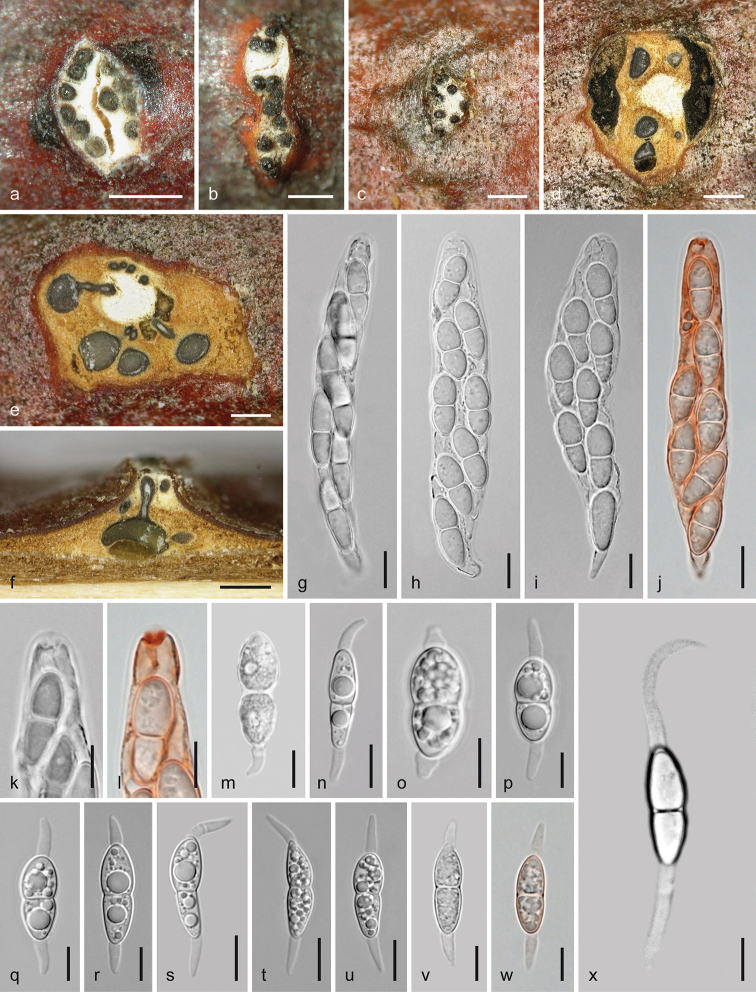
*Melanconis alni*. Sexual morph **a, b** ectostromatic discs **c** pseudostroma with ectostromatic disc in face view **d** cross section showing remnants of asexual morph at the sides of the sexual pseudostroma **e** cross section showing perithecia with lateral ostiolar necks and central column **f** vertical section showing perithecium with central ostiolar neck **g–j** asci **k, l** ascus apices showing apical ring **m–x** ascospores **j, l, w** in aqueous Congo Red **a, b, i**WU 31885 = W.J. 148 **c–f, j, o–q** epitype WU 31884 = MAIV **g, h, k, l, x**WU 37043 = J.F. 10104 **m** lectotype PC 0723592 **n**WU 37042 = D156 **r, s**WU 31882 = MAMI **t, u**WU 31883 = MAW **v**WU 31887 = W.J. 1194 **w**WU 31886 = W.J. 178. Scale bars: 400 µm (**a, b, d–f**), 500 µm (**c**), 10 µm (**g–j, n, s–u**), 7 µm (**k–m, o–r, v–x**).

**Figure 4.**
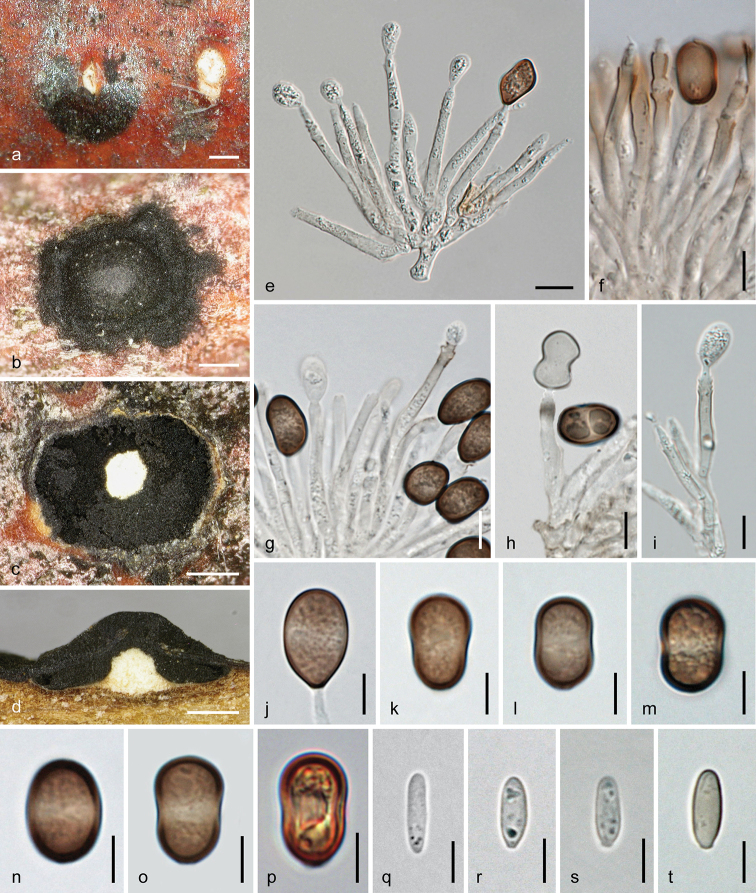
*Melanconis alni*. Asexual morph **a, b** conidiomata in face view **c** conidioma in cross section **d** conidioma in vertical section **e–i** conidiophores and conidiogenous cells **j–p** α-conidia **q–t** β-conidia **a, f**WU 31885 = W.J. 148 **b–d, h, m, q, s** epitype WU 31884 = MAIV **e, i** PC0723596 **g, j, k** lectotype PC0723592 **l, r**WU 37043 = J.F. 10104 **n, t** PC0723595 **o**WU 31886 = W.J. 178 **p***M. atrum* isotype K(M) 171588 **e–o, q–t** in 3% KOH. Scale bars: 300 µm (**a–d**), 10 µm (**e**), 7 µm (**f–i**), 5 µm (**j–t**).

##### Description.

***Sexual morph***: *Pseudostromata* developing in bark after the asexual morph and sometimes with acervuli of the asexual morph still present within their sides, 0.9–2.7 mm diam., scattered, pulvinate, more or less circular in outline, slightly projecting from the bark surface and then causing a greyish bark surface; consisting of an ectostromatic disc and perithecia embedded in an entostroma. *Ectostromatic discs* 0.3–1.4 mm diam., white to yellowish, brownish when old, flat to convex, circular, fusoid, angular or elongate in section, projecting up to 0.6 mm. *Ostiolar necks* cylindrical, laterally attached on perithecia and convergent in the disc, centrally only on centrally arranged perithecia, 1–15(–20) per disc, in the disc plane, convex to papillate and slightly projecting, with dark rounded tips; first pale brownish to greyish-brown, turning black, (70–)93–162(–210) µm (n = 33) diam. apically, mostly present at the margins but often also randomly within the disc. *Entostroma* bark-coloured, not or only slightly paler than the surrounding bark, consisting of bark cells and some light-coloured hyphae. *Perithecia* (390–)450–645(–765) µm (n = 24) diam., formed below overmature conidiomata in valsoid configuration, globose to subglobose, collapsing up- or laterally inwards upon drying. *Hamathecium* of wide multiguttulate paraphyses, collapsing, dissolving and usually absent amongst mature asci. *Asci* floating free at maturity, (68–)79–97(–110) × (10.5–)12.5–16.5(–21) µm (n = 114), narrowly clavate, fusoid, oblong to nearly ellipsoid, with an apical ring staining in Congo Red but invisible or indistinct in the strongly thickened apex in 3% potassium hydroxide (KOH), containing 8 biseriate ascospores. *Ascospores* (14.5–)16–21(–25.3) × (4.7–)6–7.8(–9) µm, l/w (1.9–)2.3–3.2(–4.8) (n = 198), hyaline, ellipsoid, clavate or inequilaterally fusoid, bicellular with upper cell usually slightly wider, slightly or strongly constricted at the median septum, thick-walled, multiguttulate or with one large and several small guttules when fresh, with a filiform, tapering and acute, less commonly short and stout rounded, triangular or truncate appendage (2.5–)4.7–10(–24.3) × (1.7–)2.3–3(– 4) µm, l/w (1–)1.8–3.8(–8.4) (n = 224) at one or both ends; in 3% KOH, appendages invisible and cells tending to be more equal.

***Asexual morph*** acervular, often conspicuous due to thick black conidial masses, first subperidermal, after ejection forming deposits 0.5–3.6 mm diam., sometimes confluent from 2–3 conidiomata and then up to 5 mm long, projecting to 0.5 mm. *Conidiomata* scattered, gregarious, sometimes confluent, pulvinate to conical, (0.6–) 0.8–2.5 mm diam., consisting of a superficial, ca. 0.2–1.3 mm wide, flat, white to yellowish, slightly projecting disc becoming concealed by dark brown to black conidial deposits, a whitish to yellowish, when old orange-brown, compact, more or less pseudoparenchymatous base, in the centre arising as central column with circular to longish outline and sometimes wavy margin, surrounded by conidiophores and black conidial chambers. *Conidiophores* emerging radially from the pseudoparenchymatous base and column surface, filiform, to ca. 50 × 4 µm, branching 1–3 times from their bases producing whorls of conidiogenous cells. *Conidiogenous cells* (10–)12–43 × 2–4 µm, annellidic, more or less cylindrical, hyaline, turning brown with age, forming more or less simultaneously two types of conidia on top. *Conidia* dimorphic, α-*conidia* (9–)10.5–12.2(–14) × (4.8–) 6.8–8(–9) µm, l/w (1.2–)1.4–1.7(–2.4) (n = 301), dark brown, more or less cuboid or subglobose and often with pinched sides or oval, oblong to broadly ellipsoid, with a diffuse or more or less well-defined, paler to subhyaline median area or stripe; β-conidia produced in small numbers, (5.3–)7.3–10.3(–11.5) × (2–)2.5–3.2(–3.7) µm, l/w (2–)2.6–3.9(–4.7) (n = 38), oblong, mostly straight, hyaline to subhyaline, turning dilute brownish with age, containing few minute guttules, with a distinct basal abscission scar.

***Culture***: Colony on CMD at 16 °C first hyaline, turning yellowish-brown from the centre, becoming covered by flocks of white aerial hyphae and conidiomata forming around the centre or colony irregular, with limited growth, turning green to black due to conidiomata; on MEA first hyaline, circular, with short aerial hyphae, forming concentric zones, the outer white, the inner turning brown, black conidiomata forming between the zones, margin becoming diffuse and the entire colony turning brown. Odour indistinct.

##### Distribution and ecology.

*Melanconis alni* occurs in Europe on dead twigs and branches of *Alnus glutinosa* and *A. incana*, mainly at lower elevations.

##### Additional material examined.

Austria, Kärnten, Eisenkappel, Bad Vellach, Vellacher Kotschna, grid square 9653/1, on *Alnus incana*, 7 Sep 1998, W. Jaklitsch W.J. 1194 (WU 31887); St. Margareten im Rosental, village area, at the brook Tumpfi, grid square 9452/4, on *Alnus glutinosa*, 18 Jul 1994, W. Jaklitsch W.J. 148 (WU 31885); Trieblach, Drau-Auen, near Kucher, grid square 9452/2, on *Alnus incana*, 7 Aug 1994, W. Jaklitsch W.J. 178 (WU 31886); Niederösterreich, Michelbach, Mayerhöfen, on *Alnus glutinosa*, 18 Jun 2011, H. Voglmayr (WU 31882, culture CBS 131693 = MAMI). FRANCE, Alpes-de-Haute-Provence, Trigance SE Castellane, at the river Jabron ca. 500 m elev. before entering the Verdon river, on *Alnus incana*, 4 Aug 2011, H. Voglmayr (WU 31884; culture MAIV); Ariége-Rimont, Peyrau, on *Alnus glutinosa*, soc. *Diplodia* sp., 26 Jul 2010, J. Fournier J.F. 10104 (WU 37043); Hauts-de-Seine, Chaville, on *Alnus glutinosa*, 11 Oct 1852, Tulasne (PC 0723589, PC 0723596); Meudon, on *Alnus glutinosa*, 13 May 1856, Tulasne (PC 0723593); Oise, Pierrefonds, on *Alnus glutinosa*, 30 Jul 1857, Tulasne (PC 0723590, PC 0723591); Yvelines, Viroflay, on *Alnus glutinosa*, July 1860, Tulasne (PC 0723594, PC 0723595); no collection data, Tulasne (PC 0723588). POLAND, S Kuligi, Biebrzański Park Narodowy, on *Alnus glutinosa*, 28 Jul 2015, H. Voglmayr (WU 37042, culture D156).

##### Notes.

*Melanconis alni* was described by Tulasne from *Alnus glutinosa* in 1856 after a presentation of the topic in April 1856. Tulasne and Tulasne (1863) validated the name in *Melanconis*, illustrated ascospores with typical long acute appendages and mentioned material from Meudon and Chaville. In PC, nine specimens of Tulasne are extant in the *Melanconis alni* folder; three of them were collected after its description in 1856 and, for one, no collection data are available. PC 0723590, PC 0723591, PC 0723593, PC 0723594 and PC 0723595 were collected after the publication date. PC 0723588 (no data) and PC 0723589, PC 0723596 from 1852 only contain asexual morph, but in the protologue, the sexual morph is also described. Therefore, we select PC 0723592, which also contains few pseudostromata of the sexual morph, as the lectotype. In PC 0723592 and PC 0723595, both α- and β-conidia are present. Generally, β-conidia are inconspicuous and produced in small numbers, i.e. they are easily overlooked. Asci in old herbarium material are shrunk and difficult to rehydrate, therefore significantly smaller than those of fresh material. In KOH, the ascus apex becomes very thick and the ring disappears; also ascospore appendages disappear in KOH.

Tulasne and Tulasne (1863) and Wehmeyer (1941) listed the following asexual morph names, amongst others, as linked to *M. alni*: *Stilbospora microsperma* Pers. Material with this name is not accessible in L; *Melanconium sphaeroideum*Link (1825) is more generally given as the name of the asexual morph. Sieber et al. (1991) used another name described by Link (1825), *Melanconium apiocarpum*, for the asexual morph of *Melanconis alni*. As Link´s type material of these taxa is not extant in B, we are unable to draw a conclusion about their identity; in addition, the descriptions in Link (1825) are vague and he gave no hosts. Therefore, we continue to use the name *M. alni*, which is generally well-known. Type material of *Melanconium atrum* Link, the generic type of *Melanconium*, described from Germany (K(M) 171588, slide from *Melanconium atrum* type material from Persoon´s herbarium) has conidia of the same shape, size and lighter median band (Fig. 4p) and may thus be conspecific with *M. alni*, but it was described from *Fagus sylvatica*. According to Sutton (1964), Link had sent his material to Persoon, because in the herbarium of the latter 3 specimens labelled *M. atrum* were extant. The host of one of these materials was identified as *Fagus*, based on bark structure. This specimen was selected as lectotype. The slide K(M) 171588 (= IMI 102914) was prepared from the lectotype and is thus an isotype. Accordingly, *Melanconium atrum* is a different species, despite its morphological similarity with *M. alni*, because the latter only occurs on *Alnus* spp. We have not seen any *Melanconium* on *Fagus*, but Petrini and Fisher (1988), Sieber et al. (1991) and Kowalski and Kehr (1992) reported and isolated *M. atrum* as an endophyte of *Fagus*. For α-conidia of isolates from *Fagus sylvatica* and *Quercus robur*, Sieber et al. (1991) reported mean sizes of 11.7–12 × 8.5–8.9 µm, which were similar to those from *Alnus glutinosa* (on average, 10.1–12.3 × 5.9–7.4 µm). However, the protein profiles revealed by isozyme electrophoresis differed markedly between the isolates from *Alnus glutinosa* and those from *Fagus*/*Quercus*, confirming them to represent distinct species that may not even be congeneric. Another fact may support the presence of morphologically similar but rare taxa on Fagaceae, as, for example, *Melanconium gourdaeforme* with similar conidia was described by Kobayashi (1968) from *Castanea*. A narrow light band is also characteristic for conidia of *Melanconiella ostryae* (Voglmayr et al. 2012).

Ascospore appendages of *Melanconis alni* may sometimes be similar to those of *M. marginalis*, at least in fractions, although truncate appendages in *M. alni* are rather a consequence of microscopic mount preparation. On *Alnus incana* both species occur, therefore the asexual morph should be sought for to reliably identify the species.

#### Melanconis betulae

Taxon classificationFungiDiaporthalesMelanconidaceae

C.M. Tian & X.L. Fan, in Fan, Du, Liang & Tian, Mycol. Progr. 15(4/40): 4 (2016).

5AEFCE1D-17E7-5177-875D-5C05A90BEE9D

##### Note.

According to Fan et al. (2016), who described this species as an asexual morph from Gansu Province in China on *Betula albosinensis*, *Melanconis betulae* can be distinguished from *M. stilbostoma* by the smaller average length of its alpha conidia (10 vs. 12 μm). Phylogenetically, *M. betulae* is remote from the other betulicolous *Melanconis* species (Fig. 1).

#### Melanconis groenlandica

Taxon classificationFungiDiaporthalesMelanconidaceae

(M. Bohn) L. Lombard & Crous, in Lombard et al. Persoonia 36: 234 (2016).

B5143FA8-EACB-5704-8D5A-FBDFCB2A0DEE

≡ Myrothecium groenlandicum M. Bohn, Mycotaxon 46: 336 (1993) (Basionym).

##### Type material.

***Holotype*** (not examined): Greenland, Qaqortoq, (isolated from) twigs of *Betula nana*, July 1991, M. Bohn (C; dried culture UPSC 3416; isotype in UPS; living cultures CBS 116450 = UPSC 3407, UPSC 3416).

##### Description

(after Bohn 1993): Colonies on PDA and MEA 30–33 mm after 10 d (52–62 mm after 20 d), appearing leathery, at first whitish to greyish, later becoming greyish-orange, particularly on MEA; margin superficial, entire on MEA but fimbriate to lobate on PDA; exudate and diffusible pigment absent; reverse greyish-orange, especially at the margin; brownish, thick-walled, chlamydospore-like swollen portions 6–18 µm diam. present. *Conidiomata* appearing after ca. 14 d as dark green pustules of various sizes, irregularly scattered over the colony surface, but sometimes arranged in concentric rings, particularly in old cultures, initially covered by mycelium but becoming almost black and shiny at later stages due to the mass of conidia; conidiomata sporodochial (acervular?), irregular, dark green, up to 2 mm diam., scattered, gregarious or coalescent, composed of a 50–70 µm high stroma of *textura intricata* and conidiophores. Marginal hyphae and setae absent. *Conidiophores* arising from the stroma, branched, septate, yellowish to brownish, ca. 40–75 µm × 2–4 µm. *Conidiogenous cells* cylindrical to subulate, 15–25 × 2–3 µm, arranged in verticils of 2–4 at the top of the conidiophore, sometimes also intercalary, provided with conspicuous, pigmented collarettes and producing conidia by percurrent growth. *Conidia* black and shiny in mass, olivaceous to brownish under the microscope, straight, cylindrical with rounded ends, sometimes slightly narrowing towards the base or apiculate, (9–)10–12(–15) × (5–)6(–7) µm, with smooth wall. Teleomorph not formed after 3 months incubation.

***Culture*** (own observations): Colony on MEA circular, first hyaline, turning and long remaining whitish, with age forming narrow concentric zones with tooth-like margins and turning pale brownish. Odour indistinct to unpleasant.

##### Distribution and ecology.

*Melanconis groenlandica* is known from North America (Greenland, USA) and Japan from *Betula maximowicziana*, *B. nana* and *B. papyrifera*.

##### Additional collections sequenced.

Japan, Hokkaido, Sorachi, Furano, Hokkaido Experimental Forest of Univ. Tokyo, on *B. maximowicziana*, 25 Sep 1964, T. Kobayashi (TFM FPH2478, culture MAFF 410219 = M4-2, ME1). USA: New Hampshire, close to the top of Mount Washington, on *Betula* sp., 28 Jul 2006, L. Mejia (BPI 879597; culture CBS 133339 = LCM 02.02 = ME13); New York, Adirondack High Peaks Region, Marcy Dam, on *Betula papyrifera*, 2 Jun 2007, L. Mejia (BPI 881485; culture CBS 133341 = LCM191.01 = ME10); ibidem, same host, 9 Jun 2007, L. Mejia (BPI 881515; culture CBS 133340 = LCM 185.01).

##### Note.

This species was isolated as a putative endophyte from *Betula nana* and described from MEA and potato dextrose agar as a species of *Myrothecium*. In our phylogenetic analyses, three isolates from North America and one from Japan grouped with the ex-type isolate of *M. groenlandica* with high support.

#### Melanconis itoana

Taxon classificationFungiDiaporthalesMelanconidaceae

Tak. Kobay., Bull. Govt Forest Exp. Stn Meguro 226: 19 (1970).

C9A1C7C8-4C22-5F69-B964-A8C79CE731AE

##### Type material.

***Holotype***: Japan, Shizuoka, Fujinomiya, Mt. Fuji, on *Betula ermanii*, 6 Aug 1968, T. Kobayashi (TFM FPH3375; ex-type culture MAFF 410080 = LFP-M4-9 = ME8).

##### Description.

See Kobayashi (1970) and Fan et al. (2016).

***Culture***: Colony on MEA circular, first hyaline, forming a white outer and brown inner zone, with radial stripes; conidiomata forming mostly in the inner zone. Odour indistinct.

##### Note.

This species occurs on *Betula ermanii* in Japan and *Betula albosinensis* in China and is particularly well characterised by its long narrow fusoid conidia, which are more or less pointed at each end. It was originally described by Kobayashi (1970) in detail and the asexual morph was redescribed by Fan et al. (2016), who gave slightly shorter measurements of conidia (12–13.5(–14) × 3.5–4(–4.5) μm). Our measurements of conidia are (13–)14.7–17.5(–20) × (3–)3.5–4.3(–4.7) µm, l/w (3–)3.6–4.7(– 5.4) (n = 100), upon examination of the holotype, which corresponds with Kobayashi (1970). The Chinese accessions genetically differ significantly from the ex-type culture from Japan (Fig. 1) and may therefore merit separation.

#### Melanconis larissae

Taxon classificationFungiDiaporthalesMelanconidaceae

Jaklitsch & Voglmayr sp. nov.

F825EFBE-AD44-5C5F-8AD3-BA5C8F3A027C

834108

Figure 5

##### Diagnosis.

*Melanconis larissae* differs from *M. stilbostoma* by α-conidia having a broad diffuse light-coloured zone.

##### Type material.

***Holotype*.** USA, New York, Adirondack Mts., Cranberry Lake, on *Betula* sp., 13 Jun 2002, L. Vasilyeva (BPI 870998; ex-type culture CBS 123196 = A.R. 3886, ME7).

##### Etymology.

Named after the collector Larissa Vasilyeva.

**Figure 5.**
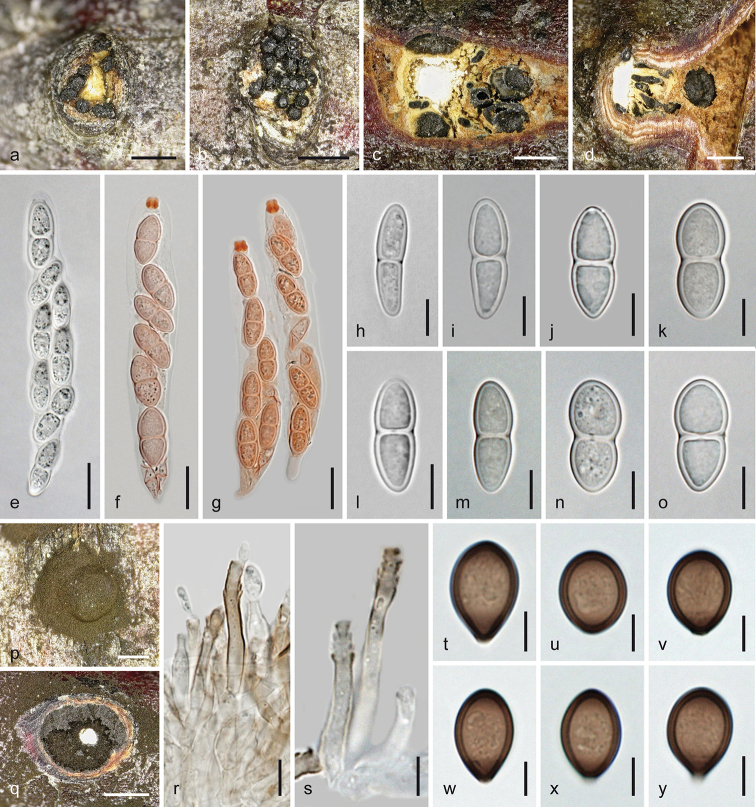
*Melanconis larissae* holotype (BPI 870998) **a–o** sexual morph **a, b** ectostromatic discs **c, d** cross sections showing white upper and yellow lower parts of central columns, ostiolar necks and perithecia **e–g** asci **h–o** ascospores **f, g** in aqueous Congo Red **p–y** asexual morph **p** conidial deposit **q** conidioma in cross section **r, s** conidiophores and conidiogenous cells (showing annellations in **s**) **t–y** α-conidia **r–y** in 3% KOH. Scale bars: 500 µm (**a–d, p, q)**, 15 µm (**e–g**), 7 µm (**h–o, r**), 5 µm (**s–y**).

##### Description.

***Sexual morph***: *Pseudostromata* 1.8–2.7 mm diam., scattered to aggregated, not or only scarcely projecting from the bark surface, pulvinate, circular to elliptical in outline; consisting of an ectostromatic disc and perithecia embedded in an entostroma around a central column and sometimes conidial locules present on the ostiolar level. *Ectostromatic discs* 0.5–1.4 mm diam. or long, slightly projecting, fusoid to circular, flat or concave, white to yellow, often concealed by ostioles; central column beneath disc brightly white at upper levels, yellow amongst ostiolar necks at lower levels, consisting of hyaline hyphae and colourless crystals. *Ostiolar necks* cylindrical, laterally or centrally attached on perithecia, convergent and irregularly inserted in the disc; visible part (88–)130–204(–230) µm (n = 32) diam., 1–12 per disc, black, subglobose to subconical with flat or pointed tips, projecting to 200 µm. *Entostroma* consisting of hyaline hyphae and bark cells. *Perithecia* (420–)490–650(–690) µm (n = 14) diam., arranged in valsoid configuration around and below central column, globose to subglobose, collapsing up- or laterally inwards upon drying. *Peridium* pseudoparenchymatous, consisting of a dark brown small-celled outer and a hyaline to brownish, large-celled inner layer. *Hamathecium* absent at maturity. *Asci* floating free at maturity, (69–)84–106(–117) × (11–)13–17.5(–19.7) µm (n = 22), fusoid to oblong, with an apical ring distinct in water and staining in Congo Red, but invisible or indistinct in 3% KOH, containing (2–)4–8 ascospores in biseriate or obliquely uniseriate arrangement. *Ascospores* (14.8–)17–21.5(–25) × (5.8–)6.5–8.3(–9.7) µm, l/w (1.9–)2.3–3(–3.7) (n = 93), ellipsoid to subfusoid, symmetric or inequilateral, bicellular, hyaline, dilute brownish when old, slightly constricted at the central to slightly eccentric septum, thick-walled, becoming verruculose with age, devoid of appendages.

***Asexual morph*** acervular, intermingled with pseudostromata of the sexual morph or developing separately, conspicuous. First white tissue (central column) forming within the bark, becoming surrounded by a yellow margin and narrow whitish to yellowish discs emerging through bark cracks, followed by the production of conidia in olivaceous to dark brown chambers. *Conidiomata* 1.3–2.7 mm diam., pulvinate, more or less circular in outline, scattered or crowded. *Covering discs* 0.25–1.2 mm long, narrowly fusoid or longish to circular, flat to convex, whitish to yellowish, becoming obscured by large, coppery to olivaceous brown conidial deposits 1–4 mm diam., projecting to 1.2 mm, also confluent from two or several conidiomata; discs and pulvinate or conical columns beneath consisting of *textura intricata* of hyaline hyphae and numerous colourless crystals, becoming brittle with age. *Conidiophores* emerging around the central column from a pseudoparenchymatous base, ca. 40–70 µm long, filiform, branched near the base and usually 1–3 fold asymmetrically at higher levels, first hyaline, turning brown from their tips; terminal *conidiogenous cells* (10.5–)14.5–28(–36.5) × (1.7–)2.5–3.5(–4.2) µm (n = 70), cylindrical and often widened towards base, with funnel-shaped collarette and up to 5 or 6 annellations, densely arranged, repetitive, producing α-conidia. *Conidia* (9.7–)11–13(–14.5) × (6.5–)7.7–9(–9.5) µm, l/w (1.1–)1.3–1.6(–2.2) (n = 66), oval, subglobose to drop-like, unicellular, dark brown, thick-walled, with a broad lighter coloured median zone and a small scar, smooth. No β-conidia detected.

***Culture***: Colony on MEA at room temperature circular, dense, first hyaline, turning rosy. Odour indistinct to musty.

##### Distribution and ecology.

*Melanconis larissae* is known from a single specimen collected in New York State from an unidentified species of *Betula*.

##### Notes.

The description of this taxon is based on a single specimen with over-mature sexual morph and well-developed asexual morph with thick masses of conidia. *Melanconis larissae* differs from *M. stilbostoma* by the broad light-coloured zone of its conidia. No β-conidia have been detected in this specimen, but oblong to ellipsoid, hyaline to dilute brownish conidia 5–9 × 1.7–5 µm, which we interpret as immature α-conidia.

#### Melanconis marginalis

Taxon classificationFungiDiaporthalesMelanconidaceae

(Peck) Wehm., Pap. Michigan Acad. I. 6: 382 (1926).

666B05B5-C5E8-5E3B-8C20-3C32A9B2E128

##### Notes.

This species is here subdivided into four subspecies below. See under subsp. marginalis for the original species.

Although Wehmeyer (1926a) combined *Diaporthe marginalis* in *Melanconis*, he later (Wehmeyer 1941) argued that the conidia only differ from those of *M. alni* in depth of pigmentation and, therefore, reduced *M. marginalis* to a subspecies of the latter. In Europe, where, owing to Wehmeyer (1941), *Melanconis* on *Alnus* was always identified as *M. alni*, Petrak (1941) reported *Melanconium dimorphum* for the first time, described both conidial types, but still found it probable that *Melanconium dimorphum* was an abnormal form of *M. sphaeroideum*, the putative name of the asexual morph of *M. alni*. Kobayashi (1970) and Jensen (1984), however, were convinced that *Melanconis marginalis* should be treated as a species separate from *M. alni*, which is here confirmed phylogenetically. In addition, ascospores of *M. marginalis* are narrower, usually more oblong and symmetric than those of *M. alni* and appendages shorter, stouter and rounded or truncate at the ends, which swell and become diffuse in mounts.

#### Melanconis marginalis subsp. europaea

Taxon classificationFungiDiaporthalesMelanconidaceae

Jaklitsch & Voglmayr subsp. nov.

3D3B8B2D-87A7-57C0-9C66-6025066F1EC7

834109

Figures 6 , 7

##### Diagnosis.

This subspecies of *Melanconis marginalis* occurs in Europe and differs from the American subsp. marginalis phylogenetically and by slightly larger asci, ascospores and ascospore appendages.

##### Type material.

***Holotype*.** Austria, Steiermark, Judenburg, Pusterwald, Hinterwinkel, grid square 8651/4, on *Alnus incana*, 11 Jun 2011, H. Voglmayr (WU 31888, culture CBS 131692 = MAI).

##### Etymology.

For its occurrence in Europe.

**Figure 6.**
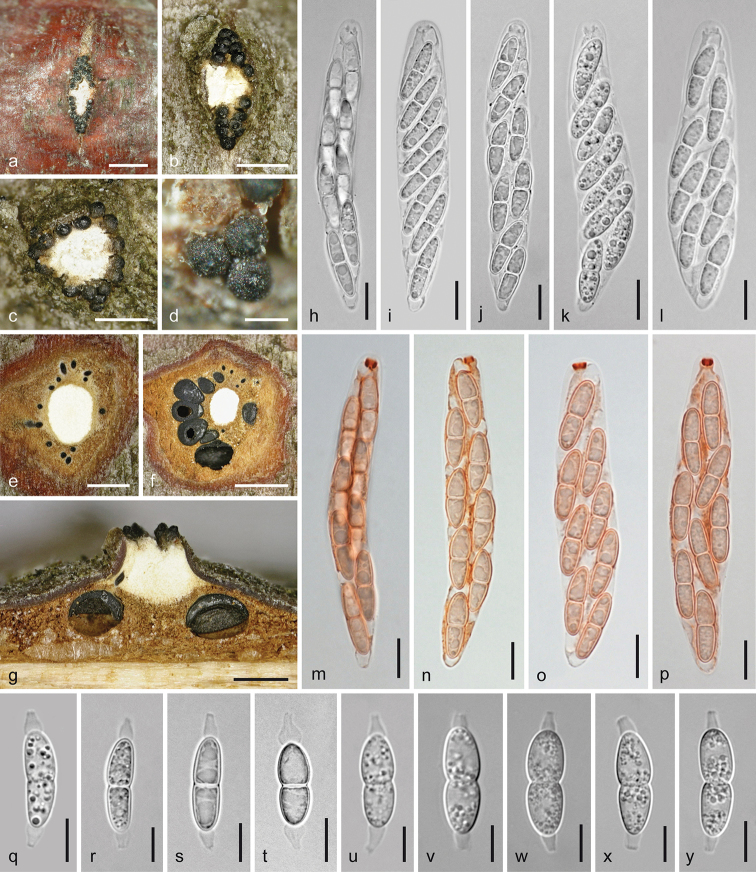
Melanconis marginalis subsp. europaea. Sexual morph **a** pseudostroma in face view **b, c** ectostromatic discs **d** subglobose visible part of ostiolar necks **e, f** cross sections (**e** showing central column and marginal ostioles **f** showing central column and perithecia) **g** vertical section showing central column and two perithecia **h–p** asci **q–y** ascospores **m–p** in aqueous Congo Red **a**WU 31890 = MAV1 **b–g, j, n, q, s, t, w–y** holotype WU 31888 = MAI **h, i, m**WU 37045 = D158 **k, r**WU 36699 **l, p**WU 31172 **o**WU 29888 **u**WU 31889 = MAV **v**WU 38243. Scale bars: 1 mm (**a, f**), 500 µm (**b, c, e, g**), 150 µm (**d**), 10 µm (**h–q, t**), 7 µm (**r, s, u–y**).

**Figure 7.**
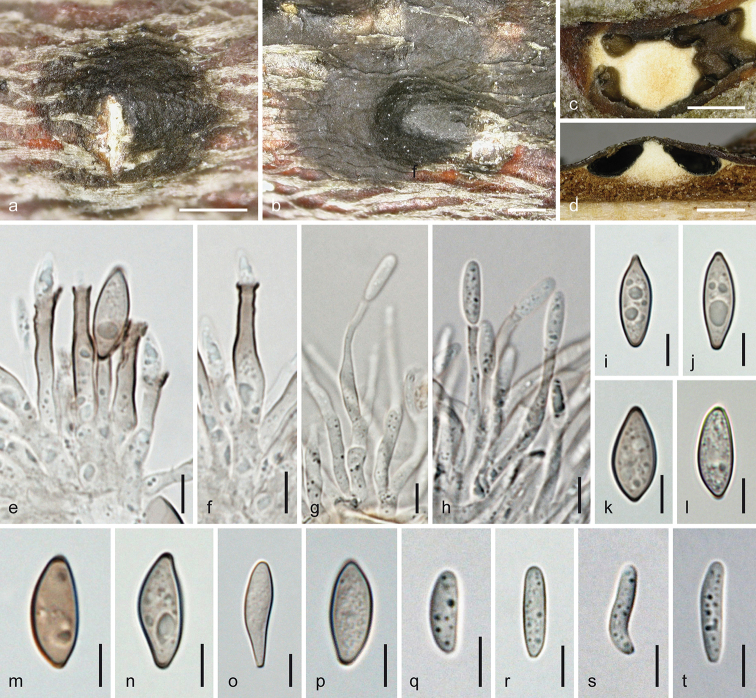
Melanconis marginalis subsp. europaea. Asexual morph **a, b** conidiomata and conidial deposits in face view **c** conidioma with β-conidia in cross section **d** conidioma with α-conidia in vertical section **e–h** conidiophores and conidiogenous cells (producing α-conidia in **e, f**, β-conidia in **g, h**) **i–p** α-conidia **q–t** β-conidia **e–t** in 3% KOH **a, b, d–f, i–k, q–s**WU 37044 = D157 **c, g, h, l, t**WU 31893 **m**WU 31891 = W.J. 1542 **n**WU 31888 = MAI **o, p**WU 31889 = MAV. Scale bars: 500 µm (**a–d**), 5 µm (**e–t**).

##### Description.

***Sexual morph***: *Pseudostromata* 1.5–3.6 mm diam., usually conspicuous and numerous, scattered to tightly aggregated, forming pustules, pulvinate, circular to elliptical in outline, typically elevated beyond bark surface; consisting of an ectostromatic disc and perithecia embedded in an entostroma around a central column. *Ectostromatic discs* 0.5–2.1 diam. or long, discrete, less commonly confluent, bright white to yellowish, turning brownish with age, variable, fusoid, elliptic or circular in outline, flat, convex, concave, entire or coarsely fissured and crumbly, projecting up to 1 mm including projecting part of the pseudostroma; central column beneath disc whitish to yellowish, consisting of hyaline hyphae and colourless crystals. *Ostiolar necks* cylindrical, laterally attached on perithecia, centrally attached only on centrally arranged perithecia, convergent in the disc margin or crowded at the ends of fusoid discs, 1–25(–35) per disc. Visible part of the ostiolar necks (53–)103–167(–212) µm (n = 90) diam., black or brown with black tips, usually circular in section, sometimes plane with the disc, but much more frequently papillate and projecting to 250 µm, often resembling minute perithecia with pointed tips or discoid with depressed centre to nearly ring-like, sometimes conical to bristle-like and projecting to 0.4 mm. *Entostroma* bark coloured, not or only slightly paler than the surrounding bark, consisting of bark cells and some light-coloured hyphae. *Perithecia* (450–)515–680(–810) µm (n = 58) diam., arranged in valsoid configuration around and below central column, globose to subglobose, collapsing up- or laterally inwards upon drying. Peridium pseudoparenchymatous, consisting of a dark brown small-celled outer and a hyaline to brownish, large-celled inner layer. *Hamathecium* of broad multiguttulate paraphyses, collapsing, dissolving and usually absent amongst mature asci. *Asci* floating free at maturity, (52–)68–85(–98) × (8.7–)10.5–15.5(–18.7) µm (n = 126), narrowly fusoid to oblong or narrowly ellipsoid, with an apical ring distinct in water and staining in Congo Red, but invisible or indistinct in 3% KOH, containing 8 biseriate or obliquely uniseriate ascospores. *Ascospores* (13.8–)17–20(–22.8) × (3.5–)4.7–6.5(–7.7) µm, l/w (2.5–)2.9–3.8(–5.5) (n = 242), hyaline, mostly oblong or narrowly ellipsoid, sometimes broadly ellipsoid upon release, symmetric or inequilateral, bicellular with nearly equal cells, slightly or strongly constricted at the median septum, multiguttulate or with few large and several small guttules when fresh, with a short and broad, rounded, sometimes tapering, angular or bell-shaped and typically terminally truncate appendage (1.8–)2.7–4.7(–8.4) × (2–)2.5–4(–5.5) µm, l/w (0.4–)0.9–1.5(–2.8) (n = 318), at one or both ends becoming invisible in 3% KOH and Congo Red after release.

***Asexual morph*** acervular, intermingled with pseudostromata of the sexual morph or more frequently developing separately, usually inconspicuous, but sometimes becoming conspicuous due to greyish-brown to dark brown conidial deposits 0.2–0.6 mm diam., rarely confluent from 2 conidiomata and then up to more than 1 cm long. First, white to yellow tissue (central column) forming within the bark, becoming visible by pustulate bark and narrow whitish to yellowish or brownish slit-like discs emerging through bark cracks, usually first followed by the production of β-conidia in olivaceous chambers and later α-conidia on the same or similar conidiophores turning the contents brown and oozing out from ends of the discs or perithecia of the sexual morph formed below the acervulus. *Conidiomata* 1–2 mm diam., pulvinate, more or less circular in outline, scattered or aggregated in lines. *Covering discs* 0.3–0.9(–1.6) mm (n = 45) long, narrowly fusoid or longish to rounded, plane to convex, becoming covered and obscured by conidial deposits; discs and pulvinate or conical columns beneath, consisting of compact *textura intricata* of hyaline hyphae and numerous colourless crystals. *Conidiophores* emerging around the central column or directly on bark in dense palisades, up to ca. 50 µm long, filiform, branched near the base or sometimes 1–2 fold asymmetrically at higher levels, hyaline, turning brown from their tips; terminal conidiogenous cells (10–)14.5–23(–27) × (1.8–)2.3–3.5(–5) µm (n = 90), cylindrical and often widened in the middle or towards base and at the funnel-shaped tips beyond its width, with up to 3 annellations, producing β-conidia and/or α-conidia. *Conidia* dimorphic, α-conidia (9–)11–14(–16.3) × (3.2–)4.5–5.5(–6.2) µm, l/w (1.7–)2.2–2.9(–3.6) (n = 172), first hyaline, soon turning pale to medium brown or greyish-brown, unicellular, mostly fusoid, but also oblong, oval or ellipsoid, straight, less commonly slightly curved, upper end usually subacute and sometimes elongated, lower end narrowly truncate, containing several guttules, smooth; β-*conidia* (8–)9–11.5(–12.7) × (2–)2.5–3(–3.3) µm, l/w (2.8–)3.3–4.6(–5.8) (n = 39), hyaline to dilute brownish, unicellular, oblong to cylindrical, straight or slightly curved, thick-walled in water, with few guttules to eguttulate, smooth.

***Culture***: Colony on CMD at 16 °C first hyaline, partly or entirely turning brownish or ochre, either covered by a dense white mat of aerial hyphae or not, sometimes becoming indistinctly zonate, sometimes forming irregularly disposed conidiomata; on MEA at room temperature, first hyaline to whitish, soon forming a few broad zones with uneven margins forming teeth, the latter partly turning brown.

##### Distribution and ecology.

Common on *Alnus alnobetula* (syn. *A. viridis*) and *A. incana* in mountainous areas of Central and Eastern Europe (confirmed for Austria, the Czech Republic fide Podlahová 1973, Romania fide Szász 1966 and Switzerland fide Sieber et al. 1991).

##### Other material examined.

Austria, Burgenland, Forchtenstein, Kohlstatt, on *Alnus incana*, 24 Sep 2016, H. Voglmayr & W. Jaklitsch (WU 37046, culture D257); Kärnten, Hüttenberg, Knappenberg, grid square 9053/3, on *Alnus alnobetula*, 10 Jun 1992, W. Jaklitsch (WU 15093); Niederösterreich, Aspangberg-St. Peter, Mariensee, grid square 8461/4, on *Alnus alnobetula*, 23 Sep 2009, H. Voglmayr (WU 29888); Steiermark, Hartberg, Pinggau, Schaueregg, Alte Glashütte, on *Alnus alnobetula*, 28 Jul 2012, W. Jaklitsch & H. Voglmayr (WU 38243); Judenburg, Pusterwald, grid square 8652/3, on *Alnus alnobetula*, 11 Jun 2011, H. Voglmayr (WU 31890, culture MAV1); Liezen, Kleinsölk, walking path between Breitlahnhütte and Schwarzensee, grid square 8649/3, on *Alnus alnobetula*, 6 Aug 2003, W. Jaklitsch W.J. 2296 (BPI 843621; culture CBS 121480 = A.R. 4013); St. Nikolai im Sölktal, Sölker Paß, grid square 8750/1, on *Alnus alnobetula*, 14 Jun 2011, H. Voglmayr (WU 31889, culture CBS 131694 = MAV); Spital am Semmering, near Pfaffensattel, grid square 8460/2, on *Alnus alnobetula*, 15 Aug 2003, W. Jaklitsch W.J. 2331 (BPI 872072; culture A.R. 4032); ibidem, same host, 8 Jul 2010, I. Krisai-Greilhuber & H. Voglmayr (WU 31172); ibidem, same host, 7 Apr 2015, H. Voglmayr (WU 36699); Tirol, Kühtai, between Haggen and Kühtai, near Zirmbachalm, grid square 8732/3, on *Alnus alnobetula*, 3 Sep 2003, W Jaklitsch W.J. 2368 (W 2004-0000062); Prägraten, Bodenalm, on *Alnus alnobetula*, 18 Jun 2015, H. Voglmayr & W. Jaklitsch (WU 37044; culture D157); Umbalfälle, grid square 8939/4, on *Alnus alnobetula*, 28 Aug 2000, W. Jaklitsch W.J. 1542 (WU 31891, BPI 748444; culture CBS 109773 = A.R. 3500; AFTOL-ID 2127); same area and host, 17 Jun 2015, H. Voglmayr & W. Jaklitsch (WU 37045; culture D158); Vienna, Landstraße, Botanical garden, Alpinum, grid square 7864/1, on *Alnus alnobetula*, 21 Aug 1994, H. Voglmayr (WU 12976); same place and host, 6 Jan 2012, H. Voglmayr (WU 31893).

##### Notes.

This subspecies differs mainly in its occurrence in (Central) Europe and by forming a clade of its own in phylogenetic analyses (Fig. 1). While the differences of the European accessions in each marker included are few, they are consistent, resulting in a well-delimited clade in the multigene analyses. As the morphological differences from M. marginalis subsp. marginalis are only small, we prefer to classify the European taxon as a subspecies rather than a separate species.

Under the name *Melanconis alni*, Podlahová (1973) described both sexual and asexual morphs of a Czech collection from *Alnus alnobetula* which clearly represents *M. marginalis*, and Szász (1966) listed and described the species (as *Melanconium dimorphum*) from Romania, again from *Alnus alnobetula*. In his isozyme studies of *Melanconium*, Sieber et al. (1991) included a Swiss isolate from *Alnus alnobetula* (as *Melanconium* sp. 1). This isolate showed a distinct but similar isozyme pattern to North American collections of *Melanconis marginalis* and had a mean conidial size of 11.7 × 4.3 µm, indicating that this isolate also represents Melanconis marginalis subsp. europaea.

#### Melanconis marginalis subsp. italica

Taxon classificationFungiDiaporthalesMelanconidaceae

(Senan., Camporesi & K.D. Hyde) Jaklitsch & Voglmayr, comb. et stat. nov.

AAD697A5-5463-5546-829D-7C87147BFD66

834110

≡ Melanconis italica Senan., Camporesi & K.D. Hyde, in Senanayake et al., Stud. Mycol. 86: 273 (2017) (Basionym).

##### Type material.

***Holotype*.** Italy, Province of Forlì-Cesena, Fiumicello di Premilcuore, on dead branch of *Alnus cordata*, 4 Dec 2013, E. Camporesi IT 1557 (MFLU 17–0879; ex-type cultures MFLUCC 16–1199, MFLUCC 17–1659; isotype BBH 42441).

##### Notes.

It is presently unclear, whether this poorly described and illustrated taxon that is only known from a single collection is simply Melanconis marginalis subsp. europaea or merits a subspecies name of its own. First, the host given by the authors, *Alnus cordata*, naturally occurs in southern Italy and Corsica and, thus, may be correct only if planted in the collection area, which is not given by the authors. Secondly, the ascospores are in the range of other subspecies and appendages are neither mentioned nor illustrated, although a few are visible in their ascus images. Apparently, ascospores were mounted in KOH, where appendages are invisible. Thirdly, they describe the asexual morph from culture and include only a poor image of conidia without giving any measurements. Last but not least, only LSU, ITS and *rpb2* are available, which are insufficient to reliably resolve its true phylogenetic position. In addition, instead of comparing their taxon with *M. marginalis*, they compare it with *M. alnicola* (Jaap 1917), which is a synonym of *Alnecium auctum*.

#### Melanconis marginalis subsp. marginalis

Taxon classificationFungiDiaporthalesMelanconidaceae

(Peck) Wehm., Pap. Michigan Acad. I. 6: 382 (1926).

90BC257D-7D5B-5002-862C-B8F41D37BC32

Figures 8 , 9

≡ Diaporthe marginalis Peck, Rep. (Annual) Trustees State Mus. Nat. Hist., New York 39: 52 (1887) [1886] (Basionym).
≡ Melanconis alni var. marginalis (Peck) Wehm., Revision of Melanconis, Pseudovalsa, Prosthecium & Titania: 27 (1941).
= Diaporthe nivosa Ellis & Everh., Proc. Acad. nat. Sci. Philad. 42: 222 (1890).
= Melanconium dimorphum Peck, Ann. Rep. New York State Mus. Nat. Hist. 40: 62 (1887).

##### Type material.

***Holotype*** of *Diaporthe marginalis*: USA, New York, Essex County, Elisabethtown, on Alnus alnobetula subsp. crispa (given as *Alnus viridis*), May 1885, C.H. Peck (NYSf 1859!; material separated into 2 envelopes NYSf 1859.1 and NYSf 1859.2). ***Epitype***, here designated: Canada, New Brunswick, Charlotte Co., 1.5 km SW of Little Lepreau, on Alnus alnobetula subsp. crispa attached to the tree, soc. *Tortilispora aurantiaca*, 3 Sep 2019, D. Malloch (WU 37850; ex-epitype cultures CBS 146200 = D321 (from ascospores), D321a (from α-conidia), D321b (from β-conidia); MBT390382).

**Figure 8.**
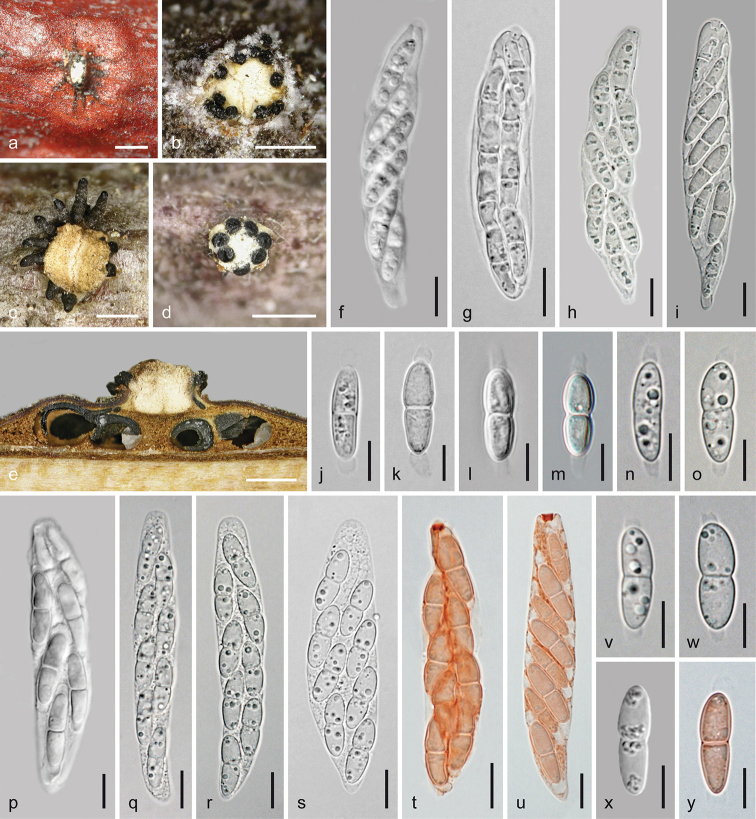
Melanconis marginalis subsp. marginalis. Sexual morph **a** pseudostroma in face view **b–d** ectostromatic discs (note conical to bristle-like ostiolar necks in **c** discoid in **d**; **e** vertical section showing central column and perithecia **f–i, p–u** asci **j–o, v–y** ascospores **t, u, y** in aqueous Congo Red **x** in 3% KOH **a, f** BPI 614844 **b, g, h, t** holotype NYSf 1859 **c, k, j** BPI 748233 **d, e, n, o, q–s, v, w, y** epitype WU 37850 **i, u** DAOM 227767 **l, m** DAOM 202917 **p** BPI 614977 **x** DAOM 86074. Scale bars: 500 µm (**a–e**), 10 µm (**f–i, q–u**), 7 µm (**j–p, v–y**).

**Figure 9.**
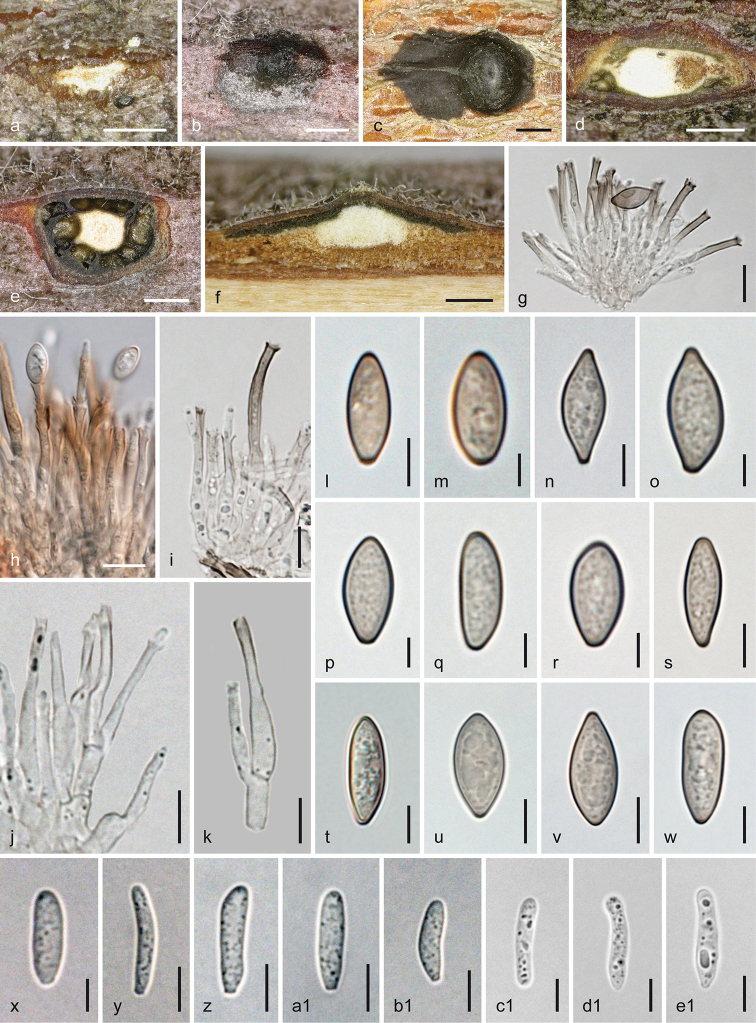
Melanconis marginalis subsp. marginalis. Asexual morph **a** early stage of covering disc **b, c** conidiomata and conidial deposits in face view **d, e** conidiomata in cross section (**d** with β-conidia, **e** with α-conidia) **f** conidioma with α-conidia in vertical section **g–k** conidiophores and conidiogenous cells (producing α-conidia in **g, h** β-conidia in **i–k**) **l–w** α-conidia **x–e1** β-conidia **g–e1** in 3% KOH **a, b, d–g, i–k, n–s, x–b1** epitype WU 37850 = D321 **c, t–w, c1–e1** DAOM 227767 **h, l, m** BPI 614844. Scale bars: 300 µm (**a, e, f**), 500 µm (**b, d**), 1 mm (**c**), 10 µm (**g, h**), 7 µm (**i, t–v**), 5 µm (**j–l, n, s, w, y–e1**), 3 µm (**m, o–r, x**).

##### Description.

***Sexual morph***: *Pseudostromata* immersed in bark causing pustules, scattered or aggregated, sometimes fused in pairs, 1.2–3.2 mm diam., pulvinate, circular to elliptic in outline, often elevated beyond bark surface; consisting of an ectostromatic disc and perithecia embedded in an entostroma around a central column, sometimes also acervuli containing α-conidia on the ostiolar level. *Ectostromatic discs* 0.3–1.5(–2) mm diam. or long, bright white to yellowish or cream, flat, convex or concave, sometimes fissured or with dark stellate stripes around disc on the bark surface, sometimes concealed by ostioles, circular, elliptic or fusoid in outline, typically distinctly projecting up to 1 mm including projecting part of the pseudostroma; central column beneath disc white to yellowish, consisting of hyaline hyphae and colourless crystals. *Ostiolar necks* cylindrical, laterally attached on perithecia, centrally attached only on centrally arranged perithecia, convergent in the disc margin or crowded at the ends of fusoid discs, sometimes completely filling disc, 1–15(–22) per disc. Visible part of the ostiolar necks (55–)87–153(–230) µm (n = 128) diam., shiny black or brown with black tip, flat discoid to ring-like, papillate to subglobose with pointed tip or conical, sometimes bristle-like and projecting up to 0.6 mm. *Entostroma* bark coloured, not or only slightly paler than the surrounding bark, consisting of bark cells and some light-coloured hyphae. *Perithecia* (420–)480–650(–750) µm (n = 34) diam., arranged in valsoid configuration around and below central column, depressed subglobose, collapsing up- or laterally inwards upon drying. Peridium pseudoparenchymatous, consisting of a dark brown small-celled outer and a hyaline to brownish, large-celled inner layer. *Hamathecium* of broad multiguttulate paraphyses, collapsing, dissolving and usually absent amongst mature asci. *Asci* floating free at maturity, (46–)56–69(–82) × (10–)11–14.5(–18) µm (n = 116), mostly oblong to fusoid, but also clavate or narrowly ellipsoid, with an apical ring distinct in water and staining in Congo Red but invisible or indistinct in 3% KOH, containing 8 ascospores in bi- or obliquely uniseriate arrangement. *Ascospores* (13.8–)15.5–18(–20.7) × (3.7–)4.5–5.7(–7.7) µm, l/w (2.4–)2.9–3.7(–4.4) (n = 236), hyaline to yellowish, oblong to ellipsoid, bicellular with equal or slightly unequal cells, slightly to distinctly constricted at the more or less median septum, multiguttulate or with few large and several small guttules when fresh, with a roundish to triangular or broadly oblong to beak-like and truncate appendage (1.1–)1.8–3.5(–6.1) × (2.2–)2.5–3.5(–4.2) µm, l/w (0.4–)0.6–1.2(–2.1) (n = 140) at each end; in 3% KOH, ascospores wider and more ellipsoid; appendages mostly invisible.

***Asexual morph*** acervular, intermingled with pseudostromata of the sexual morph or more frequently developing separately, usually inconspicuous but sometimes becoming conspicuous due to greyish-brown to dark brown conidial deposits to 2.7 mm diam., sometimes confluent from 2 conidiomata and then up to 7 mm long. First white to yellow tissue (central column) forming within the bark, becoming visible by pustulate bark and narrow whitish to yellowish or brownish slit-like discs emerging through bark cracks, usually first followed by the production of β-conidia in olivaceous chambers, followed by fusion of the chambers and production of α-conidia on the same or similar conidiophores, turning the cavity brown and oozing out from ends of the discs or perithecia of the sexual morph formed beneath. *Conidiomata* ca. 0.9–3 mm long or diam., pulvinate, more or less circular in outline, scattered or aggregated in lines. *Covering discs* 0.3–0.7 mm long or diam., narrowly fusoid or longish to circular, plane to convex, white-yellowish-brownish, becoming covered and obscured by conidial deposits; discs and pulvinate or conical columns beneath consisting of compact *textura intricata* of hyaline hyphae and numerous colourless crystals. *Conidiophores* emerging around the central column from a *textura intricata*, fasciculate, filiform, branched near the base or sometimes 1–2 fold asymmetrically at higher levels, hyaline, turning brown from their tips; terminal conidiogenous cells (10–)13.5–23(–31) × (1.7–)2–3(–3.5) µm (n = 68), cylindrical and often widened in the middle or towards the base and at the funnel-shaped tips beyond its width, annellidic, producing α- and/or β-conidia. *Conidia* dimorphic, α-conidia (9–)10.5–13.3(–16.8) × (3.8–)4.5–5.3(– 6) µm, l/w (1.7–)2–2.8(–3.9) (n = 171), first hyaline, soon turning light to medium brown, unicellular, mostly fusoid, but also oblong, oval, citriform or ellipsoid, straight or slightly curved to sigmoid, upper end often subacute, lower end narrowly truncate, containing several guttules or eguttulate, smooth; β-conidia (6–)8–10.5(–12.2) × (1.7–)2.2–2.8(– 3) µm, l/w (2.4–)3–4.6(–6.4) (n = 46), hyaline to dilute brownish, unicellular, oblong to cylindrical, sometimes reniform, straight or curved, thick-walled in water, with few guttules to eguttulate, smooth.

***Culture***: Colony on CMD at 22 °C circular with slightly uneven margin, hyaline to whitish, forming a broad inner white zone with tooth-like margin and narrow hyaline outer zones; on MEA at room temperature circular, first hyaline to white, margin becoming diffuse, narrow or coarse concentric zones formed, turning brown from the margins, aerial hyphae short, dense, surface sometimes becoming imbricate, sometimes growth limited and ceasing after a few weeks.

##### Distribution and ecology.

Widespread in North America and also occurring in Japan and eastern Russia on various subspecies of *Alnus alnobetula* and *A. incana*; recorded also from *A. rubra* (Sieber et al. 1991; see also material cited below).

##### Additional material examined.

Canada, British Columbia, Kelowna, June Springs road, June Springs trail, on *Alnus incana*, 18 Jul 1999, J. Ginns 10834 (DAOM 227767; measurements separately given, see below under Notes); Nelson, on Alnus incana subsp. tenuifolia, soc. *Cryptosporella* sp., 26 Jun 1930, G.G. Hedgcock (BPI 614844, F.P. 50704); Victoria, Lake Cowichan, Mesachie Lake, 48.7942N 124.1573W, on *Alnus rubra*, 14 Sep 1988, C. Dorworth (DAVFP 24976, dried culture PFC-051 only); Victoria, Ucluelet, Kennedy Lake, 49.0416N 125.5315W, on *Alnus rubra*, 16 May 1987, C. Dorworth (DAVFP 24972, dried culture PFC-025 only); Manitoba, W Hawk Lake, on *Alnus* sp., 5 Jun 1932, G.R. Bisby 4593 (DAOM 202917); Nova Scotia, Kings Co., Glenmont, on Alnus alnobetula subsp. crispa (as Alnus crispa var. mollis), 25 Jul 1936, I.L. Conners (Ottawa 3798 (DAOM)); Kentville, on Alnus alnobetula subsp. crispa, 11 May 1953, D. Creelman (DAOM 54346); Ontario, District of Nipissing, Temagami Forest Reserve, Lake Temagami, Bear Island, on Alnus alnobetula subsp. crispa (as Alnus viridis var. mollis), 19 Jun 1933, R.F. Cain 2686 (DAOM 86075); trail at Matagama Point, on Alnus alnobetula subsp. crispa (as Alnus crispa var. mollis), 23 Jun 1933, R.F. Cain 2687 (DAOM 86074); Sharp Rock Inlet, on Alnus alnobetula subsp. crispa (as Alnus crispa var. mollis), 29 Jun 1933, R.F. Cain (BPI 614977, F.P. 69748). Japan, Hokkaido, Shirikinai, on Alnus alnobetula subsp. maximowiczii, 1 Sept 1967, T. Oguchi (TFM FPH3290; culture MAFF 410218 = M4-6, ME9). RUSSIA, Sakhalin Island, Lake Dvoynoe, on Alnus alnobetula subsp. maximowiczii, 3 Aug 2000, A. Bogachova, comm. L. Vasilyeva (BPI 748233; culture CBS 109496 = A.R. 3529, ME2). USA, Alaska, Fairbanks, Large Animal Research Station, on *Alnus alnobetula*, 5 Aug 2011, L. Mejia (BPI 884096; culture A.R. 4864, ME5); same area, on *Alnus alnobetula* (given as *Betula neoalaskana*), 5 Aug 2011, L. Mejia (BPI 884097; culture CBS 133346 = A.R. 4865, ME6); Juneau, on Alnus alnobetula subsp. sinuata, 6 Sep 1936, D.V. Baxter (BPI 615125).

##### Notes.

The asexual morph of Melanconis marginalis subsp. marginalis is inconspicuous with usually only thin greyish patches of α-conidia. The two types of conidia may be present at the same time or only one is present; acervuli containing α-conidia are sometimes present in pseudostromata of the sexual morph. The specimen DAOM 227767 from *Alnus incana* differs from all others by very large and conspicuous conidial deposits (Fig. 9c), slightly larger α-conidia, (13–)14.5–16.5(–17.5) × (5–)5.8–7(–8) µm, l/w (1.8–)2.1–2.8(–3.4) (n = 70) and longer and more slender β-conidia, (7.5–)12.5–16(–17.3) × (1.7–)2.2–3(–3.5) µm, l/w (4–)4.6–6.7(–9) (n = 35) and also by slightly larger asci, (68–)74–88(–95.5) × (10–)12–15.5(–18.2) µm (n = 26), which approach the European subspecies. Although Jensen (1984) gave a range of 9–17 × 3–7 µm for α-conidia and 10–18 × 2–3 µm for β-conidia of *M. marginalis*, it is unclear, whether all examined specimens, including DAOM 227767, phylogenetically belong to M. marginalis subsp. marginalis or a different subspecies or even species. Jensen (1984) reported exceptionally long ascospores (19–32 µm) for four of his collections from Idaho, which also differed in their colony characters; due to lack of DNA data, the taxonomic status of these collections is unclear. While all our North American and Eastern Asian accessions of M. marginalis subsp. marginalis sequences originated from various subspecies of *Alnus alnobetula*, the accessions investigated by Jensen (1984) originated from Alnus incana subsp. tenuifolia. Sieber et al. (1991), who investigated *M. marginalis* from British Columbia, recorded mean conidial sizes of 11.2–11.8 × 4.4–4.7 µm for two isolates from *A. rubra*, while those from three isolates of *Alnus alnobetula* were slightly larger (13.6–14 × 5.6–5.9 µm). These data demonstrate the need of additional detailed investigations of the *M. marginalis* complex in western North America. Kobayashi (1970) determined the following sizes for Japanese collections of *M. marginalis*: asci 70–93 × 10–15 µm, ascospores 15–23 × 4–6.5 µm, mostly 17–20 × 4.5–5.5 µm, α-conidia 11.5–15 × 4–6.5 µm, β-conidia 7.5–12.5 × 1.5–2.5 µm. He also mentioned that the Japanese collections usually lacked ascospore appendages, which, however, may be due to the use of a mounting medium instead of water in his microscope mounts. This is supported by the fact that he also reported a lack of appendages in his *M. pterocaryae*, which was disproved by re-investigation of the type (Voglmayr et al. 2017).

Sizes of asci depend on the age of the material. They shrink with time and in specimens, which are 20 or more years old, they are smaller and do not obtain the original size even in KOH; also, it is very difficult to release ascospores from asci. In fresher specimens, asci are easily separable and ascospores are readily released. Vital asci open readily in mounts. Nonetheless, fresh asci of the epitype of subsp. marginalis were distinctly smaller than fresh asci of subsp. europaea.

Poor representation of the asexual morph in fungarium specimens may be due to the fact that the sexual morph is usually abundant, with numerous white ectostromatic discs; thus, the asexual morph may have been neglected during collecting or even discarded. β-conidia are often absent or scant and old amongst α-conidia in dark conidial deposits, hence they are either not formed or produced before α-conidia.

#### Melanconis marginalis subsp. tirolensis

Taxon classificationFungiDiaporthalesMelanconidaceae

Jaklitsch & Voglmayr subsp. nov.

A68202E1-7A05-5A20-949B-1D27F9B9545F

834111

Figures 10 , 11

##### Diagnosis.

This subspecies differs from Melanconis marginalis subsp. europaea and subsp. marginalis phylogenetically and by slightly larger α-conidia, asci, ascospores and ascospore appendages.

##### Type material.

***Holotype***: Austria, Tirol, Osttirol, Prägraten am Großvenediger, Umbalfälle, grid square 8939/4, on *Alnus alnobetula*, 10 Sep 2001, W. Jaklitsch W.J. 1796 (BPI 872035; ex-type culture CBS 122310 = A.R. 3748 = ME4; part preserved as isotype WU 31892, asexual morph only present in the latter).

##### Etymology.

Named after its occurrence in Tirol, Austria.

**Figure 10.**
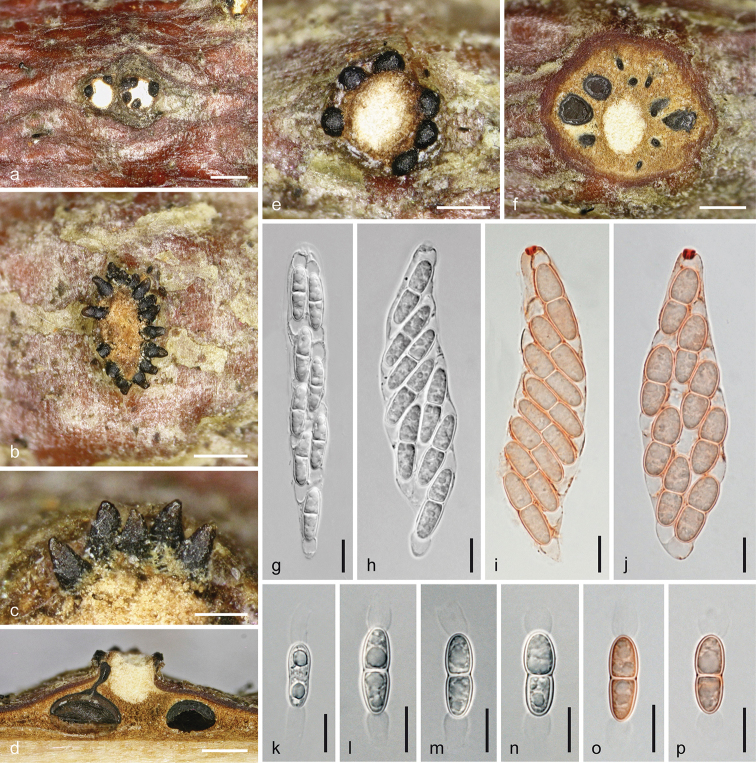
Melanconis marginalis subsp. tirolensis. Sexual morph **a, b** pseudostromata with ectostromatic discs **c** conical ostioles **d** vertical section showing central column and two perithecia **e** ectostromatic disc with subglobose ostiolar tips **f** cross section showing central column, marginal ostioles and upper parts of perithecia **g–j** asci (compressed in **j**) **k–p** ascospores; **i, j, o, p** in aqueous Congo Red **a, c, k–p** holotype BPI 872035 **b, d–j** isotype WU 31892. Scale bars: 500 µm (**a, b, d, f**), 150 µm (**c**), 300 µm (**e**), 10 µm (**g–p**).

**Figure 11.**
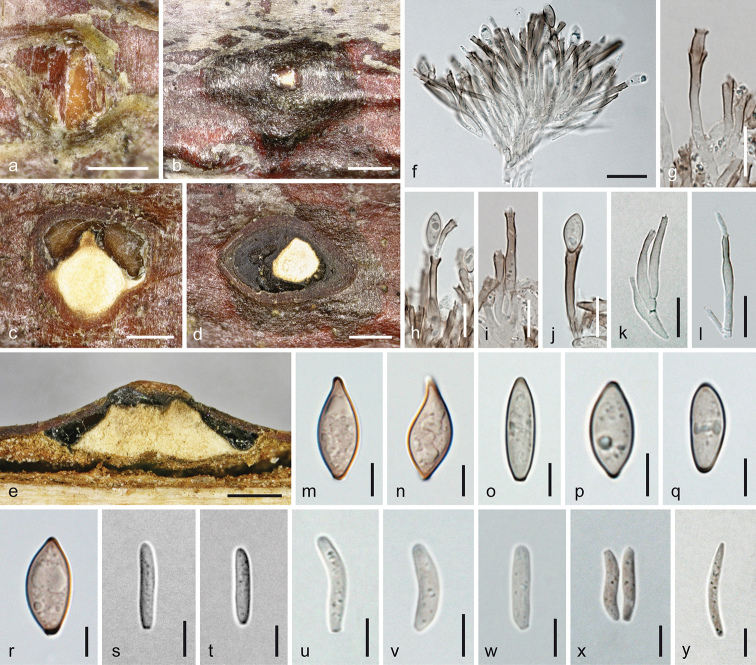
Melanconis marginalis subsp. tirolensis (isotype WU 31892). Asexual morph **a, b** conidiomata showing covering discs in face view **c, d** conidiomata in cross section (**c** with β-conidia **d** with α-conidia) **e** conidioma with α-conidia in vertical section **f–l** conidiophores and conidiogenous cells (**k, l** producing β-conidia) **m–r** α-conidia **s–y** β-conidia **f–y** in 3% KOH. Scale bars: 500 µm (**a–e**), 15 µm (**f**), 10 µm (**g–l**), 5 µm (**m–y**).

##### Description.

***Sexual morph***: *Pseudostromata* 1.3–5.5 mm diam., conspicuous and numerous, scattered to aggregated, pulvinate, circular to elliptical in outline, elevated beyond bark surface forming pustules; consisting of an ectostromatic disc and perithecia embedded in an entostroma around a central column. *Ectostromatic discs* 0.35–1.55 mm (n = 43) diam. or long, bright white to yellowish, turning brownish with age, mostly fusoid, also elliptic or circular in outline, mostly flat, crumbly, distinctly projecting up to 1.3 mm, including projecting part of the pseudostroma; central column beneath disc white to yellowish, consisting of hyaline hyphae and colourless crystals. *Ostiolar necks* cylindrical, laterally or centrally attached on perithecia, convergent in the disc margin or crowded at the ends of fusoid discs, 1–15 per disc. Visible part of the ostiolar necks (53–)85–180(–240) µm (n = 56) diam., black, often with olivaceous tips, frequently conical to bristle-like and projecting to 0.4 mm, but also papillate, resembling minute perithecia or discoid with depressed centre. *Entostroma* bark coloured, not or only slightly paler than the surrounding bark, consisting of bark cells and some light-coloured hyphae. *Perithecia* (510–)570–780(–900) µm (n = 36) diam., arranged in valsoid configuration around and below central column, globose to subglobose, collapsing up- or laterally inwards upon drying. Peridium pseudoparenchymatous, consisting of a dark brown small-celled outer and a hyaline to brownish, large-celled inner layer. *Hamathecium* absent at maturity. *Asci* floating free at maturity, (74–)86–102(–115) × (11.3–)13–20(–25) µm (n = 61), fusoid to oblong or clavate, short-stipitate prior to full maturation, with an apical ring distinct in water and staining in Congo Red, but invisible or indistinct in 3% KOH, containing 8 biseriate or obliquely uniseriate ascospores. *Ascospores* (15.8–)17.8–21.2(–24) × (4.5–)5.5–7(–8) µm, l/w (2.5–)2.8–3.5(–4) (n = 123), hyaline, turning pale brown with age, oblong to ellipsoid, symmetric to slightly inequilateral with nearly equal cells, slightly or strongly constricted at the median septum, multiguttulate or with 1–2 large and several small guttules when fresh, with a short and broad, rounded, parabolic or vesicular, sometimes tapering but typically terminally broadly truncate appendage (2–)3.8–6.2(–9.5) × (3–)4–5.7(–7.2) µm, l/w (0.4–)0.8–1.4(–2) (n = 104) at each end, after release becoming invisible in 3% KOH, but partly persistent in Congo Red.

***Asexual morph*** acervular, intermingled with pseudostromata of the sexual morph or developing separately, inconspicuous. First white to yellowish tissue (central column) forming within the bark, becoming visible by slightly pustulate bark and narrow whitish to yellowish discs emerging through bark cracks, usually first followed by the production of β-conidia in olivaceous chambers and later α-conidia or both more or less simultaneously on the same or similar conidiophores, chambers fusing into a single locule, turning brown and dark conidial patches 0.5–1.5 mm diam. or perithecia of the sexual morph forming. *Conidiomata* 1.2–3.2 mm diam., pulvinate, more or less circular in outline, scattered or crowded. *Covering discs* 0.2–1.5 mm (n = 14) diam. or long, narrowly fusoid or longish to circular, flat to convex, whitish, yellowish to brownish; discs and pulvinate or conical columns beneath consisting of compact *textura intricata* of hyaline hyphae and numerous colourless crystals, becoming brittle with age. *Conidiophores* emerging around the central column in dense palisades, up to ca. 65 µm long, filiform, branched near the base and usually 1–3 fold asymmetrically at higher levels, first hyaline, turning brown from their tips; terminal conidiogenous cells (9–)15–25(–28) × (1.7–)2.3–3.2(–3.7) µm (n = 63), cylindrical and often widened towards base, even wider at the funnel-shaped tips, with up to 3 annellations, proliferating and producing α- or β-conidia. *Conidia* dimorphic, α-conidia (10–)11.5–16.3(–21.8) × (2.5–)4.5–6.3(–7.5) µm, l/w (1.8–)2.1–3.2(–5.3) (n = 70), first hyaline, soon turning light to medium brown, mostly fusoid, also oblong, oval or ellipsoid, straight or slightly curved, upper end usually subacute and sometimes elongated, lower end narrowly truncate, containing several guttules, smooth; β-conidia (7.3–)8.8–12(–16.5) × (2–)2.2–2.7(–3.4) µm, l/w (2.6–)3.3–5.3(–8.9) (n = 104), hyaline, dilute brownish with age, sometimes turning rosy in 3% KOH, oblong to cylindrical, straight or curved or sigmoid, thick-walled in water, smooth, with truncate basal scar and minute guttules to eguttulate.

***Culture***: Colony on MEA dense, first hyaline to white, with restricted growth, forming brown radial portions mostly submerged in the agar. Odour unpleasant.

##### Distribution and ecology.

Co-occurring with Melanconis marginalis subsp. europaea in a subalpine area of eastern Tyrol, Austria, Europe, on *Alnus alnobetula*.

##### Additional material examined.

Austria, Tirol, Osttirol, Virgental, Prägraten am Großvenediger, Lasörling, Zopatnitzen on path between Wetterkreuz and Berger See, 2100 m a.s.l., on *Alnus alnobetula*, 26 Oct 2019, H. Voglmayr & C.M. Botoaca (WU 37851; culture D322a (from α-conidia)).

##### Notes.

As this subspecies differs morphologically only subtly from the other varieties of *M. marginalis*, we prefer to classify it as a subspecies rather than a separate species. While the ITS sequences of Melanconis marginalis subsp. tirolensis differs from Melanconis marginalis subsp. europaea in only a single base pair, the differences are substantial in all other markers included, particularly *tef1* and *tub2*.

#### Melanconis pacifica

Taxon classificationFungiDiaporthalesMelanconidaceae

Jaklitsch & Voglmayr sp. nov.

5533B67C-06C9-547A-84AB-FA8223488A9B

834112

Fig. 12

##### Diagnosis.

This species is characterised by its occurrence on *Alnus rubra* and α-conidia, which are wider and darker than those of *M. marginalis* and differ by a different shape and absence of a light band from those of *M. alni*.

##### Type material.

***Holotype.*** Canada, British Columbia, Sidney, off Jura, on *Alnus rubra*, 26 May 2000, M.E. Barr 1021A (DAOM 230637; ex-type culture CBS 109744; isotype BPI 748446).

##### Etymology.

For its occurrence in the Pacific region of western North America.

**Figure 12.**
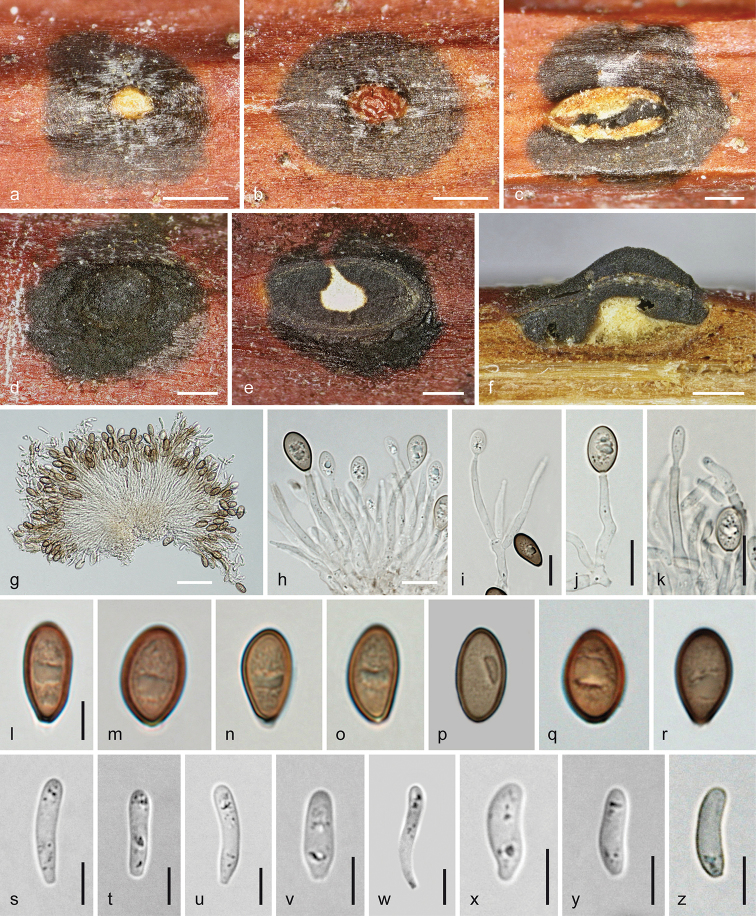
*Melanconis pacifica*. Asexual morph **a–d** conidiomata in face view **e** conidioma in cross section **f** conidioma in vertical section **g–k** conidiophores (**g** with both conidial types, note annellations in right conidiophore in **k**) **l–r** α-conidia **s–z** β-conidia **a–k, n–p, z** DAOM 220988 **l, m, r–y** holotype DAOM 230637 **q** isotype BPI 748446 **g–o, r–z** in 3% KOH. Scale bars: 300 µm (**a–f**), 30 µm (**g**), 10 µm (**h–k**), 5 µm (**l–z**).

##### Description.

***Asexual morph***: *Conidiomata* 0.7–2.1 mm diam., visible as dark brown to blackish spots, acervular, subperidermal, scattered, discrete, rarely two confluent, pulvinate to conical, consisting of an erumpent central or eccentric, circular or elliptic to fusoid, flat or convex *disc* 0.2–1.3 mm diam., whitish, yellowish to reddish-orange when young, becoming concealed by ejected conidia and internally a narrow central or eccentric, whitish to yellowish stromatic column sometimes fraying out laterally and a dark ring-like periphery containing conidia. *Conidia* becoming discharged through a mostly slit-like rupture of the disc, forming dark brown to black, up to 0.7 mm high masses or tendrils. *Conidiophores* densely aggregated forming palisades, up to ca. 50 µm long, arising from a yellowish, nearly pseudoparenchymatous tissue of compacted hyphae, either consisting solely of conidiogenous cells or of a stout main axis with few side branches and a terminal whorl of 2–4 more or less vertical conidiogenous cells, hyaline to yellowish. *Conidiogenous cells* mostly 11–32 × (2–)2.5–3.3(–3.5) μm, annellidic, more or less cylindrical, hyaline, turning brown with age, forming simultaneously two types of conidia on top. *Conidia* dimorphic, α-*conidia* (8.8–)10.5–12.5(–15.5) × (5–)6.5–7.7(–8.8) µm, l/w (1.2–)1.4–1.8(–2.7) (n = 615), oval to ellipsoid, dark brown, with a distinct basal abscission scar; β-*conidia* (6.2–)8.2–12.5(–18.5) × (2–)2.3–3(–3.6) µm, l/w (1.7–)3–4.9(–7.6) (n = 103), oblong to cylindrical, straight or curved, sometimes sigmoid or kidney-shaped to subellipsoid, hyaline, turning dilute brownish with age, typically containing two subterminal groups of minute guttules, with a distinct basal abscission scar.

***Culture***: Colony on MEA circular, first hyaline, turning white and later brownish in spots or patches, with stellate margin and radial stripes; black conidiomata forming along the stripes. Odour indistinct.

##### Additional materials examined

(all on/from *Alnus rubra*). Canada, British Columbia, Sidney, Bazan Bay, 28 May 1995, M.E. Barr (DAOM 220988); Victoria, 26 km N of Campbell River, 50.1262N, 125.3084W, 2 Jan 1989, T.N. Sieber (DAVFP 24981, dried culture PFC-071 only); Caycuse, W shore of Cowichan Lake, 48.8810N, 124.4321W, 24 Oct 1988, T.N. Sieber (DAVFP 24980, dried culture PFC-068 only); Gordon Head, C. Dorworth’s property, 48.4396N, 123.3380W, 4 Jun 1988, C. Dorworth (DAVFP 24973, dried culture PFC-043 only); East Sooke, 48.4377N, 123.7436W, 29 Jun 1948, W.G. Ziller (DAVFP 3092); Nanaimo, DeCourcy Island, 49.0641N, 123.7732W, 1 Jun 1988, C. Dorworth (DAVFP 24974, dried culture PFC-047 only); Parksville, NW Bay, 3.1 km W of M&B office, 49.3238N, 124.1479W, 13 Jul 1988, C. Dorworth (DAVFP 24975, dried culture PFC-050 only); Port Renfrew, Sombrio Beach, 48.5229N, 124.2866W, 4 Nov 1988, C. Dorworth (DAVFP 24977, dried culture PFC-053 only); Revelstoke, Jordan River, gravel pit S of the river, 48.4356N, 124.0140W, 24 Oct 1988, C. Dorworth (DAVFP 24978, dried culture PFC-055 only); ibid., 24 Oct 1988, T.N. Sieber (DAVFP 24979, dried culture PFC-067 only); Sooke, East Sooke Park, Babbington Trail, 48.3485N, 123.6073W, 9 Sep 1988, C. Dorworth (DAVFP 25029, dried culture PFC-054).

##### Notes.

The description is largely based on DAOM 220988 due to good development of conidiomata. However, we select DAOM 230637 as the holotype, because DNA data are only available for this specimen. Microscopic data of the two specimens are identical. This species is currently only known as an asexual morph. One specimen from Victoria (DAVFP 3092) contains also an immature sexual morph, which corresponds to *Melanconis alni* superficially. Barr apparently identified her collections as *M. marginalis* because the latter was, at that time, considered to be the only alnicolous species occurring in North America (Jensen 1984), which also occurs on *A. rubra* (Sieber et al. 1991). However, the conidia of the latter species are longer, more fusoid, have a larger l/w ratio and are lighter in colour than those of *M. pacifica*. α-conidia of *M. pacifica* and *M. alni* are virtually identical in size. Those of the latter, however, have a different shape, a median light band and a more greyish-brown colour. Remarkably, Wehmeyer (1941) mentioned a collection from the American Pacific region (Oregon) which had conidia resembling *Melanconium sphaeroideum*, a synonym of *M. alni*. Sieber et al. (1991) included 10 isolates from *Alnus rubra*, sampled in British Columbia, that they identified as *Melanconium apiocarpum*, another synonym of *M. alni* (see above), based on conidial size and shape. Their measurements and, in particular, their illustration (fig. 2a) of α-conidia fully agree with *M. pacifica*. The isozyme patterns of Sieber et al. (1991) revealed high similarities, but also diagnostic differences between the isolates from European *A. glutinosa* and Canadian *A. rubra*, which is in agreement with the close phylogenetic relationship between *M. alni* and *M. pacifica*. Our morphological re-investigations of the isolates of Sieber et al. (1991), which are kept as dried cultures at DAVFP (see specimens cited above), confirmed that they represent *M. pacifica*.

In DAOM, two additional specimens, labelled *Melanconis marginalis* collected by Barr in the same area, are extant, DAOM 227727 and DAOM 227345. These specimens, however, do not contain *M. pacifica*, but the sexual morph of a *Diaporthe* sp.

#### Melanconis stilbostoma

Taxon classificationFungiDiaporthalesMelanconidaceae

(Fr. : Fr.) Tul. & C. Tul., Select. fung. carpol. (Paris) 2: 115 (1863).

22504CDE-F072-5268-B981-F3B1A0C13B67

Figure 13

≡ Sphaeria stilbostoma Fr. : Fr., K. svenska Vetensk-Akad. Handl., ser. 3, 39: 102 (1818) (Basionym)
≡ Melanconis stilbostoma (Fr. : Fr.) Tul., Annls Sci. Nat., Bot., sér. 4, 5: 109 (1856). (Nom. inval., Art. 35.1).
?= Melanconium bicolor Nees : Fr., Syst. Pilze (Würzburg): 32 (1816) [1816–17].
= Melanconium betulinum J.C. Schmidt & Kunze, Deutschl. Schwämme, Neunte Lieferung: 3 (1819).
= Melanconium elevatum Corda, Icon. fung. (Prague) 3: 22 (1839).

##### Type material.

***Lectotype.*** Sweden, without data, Fries, Scleromyc. Suec. no. 145, as *Sphaeria stilbostoma* (UPS:BOT:F-117590, lectotype here designated; MBT390467)). ***Epitype***, here designated: Austria, Tirol, Prägraten, Umbalfälle, grid square 8939/4, on *Betula pendula*, 28 Aug 2000, W. Jaklitsch W.J. 1543 (BPI 748447; ex-epitype culture CBS 109778 = A.R. 3501 = ME11; AFTOL-ID 936; MBT390383; iso-epitype WU 31897).

**Figure 13.**
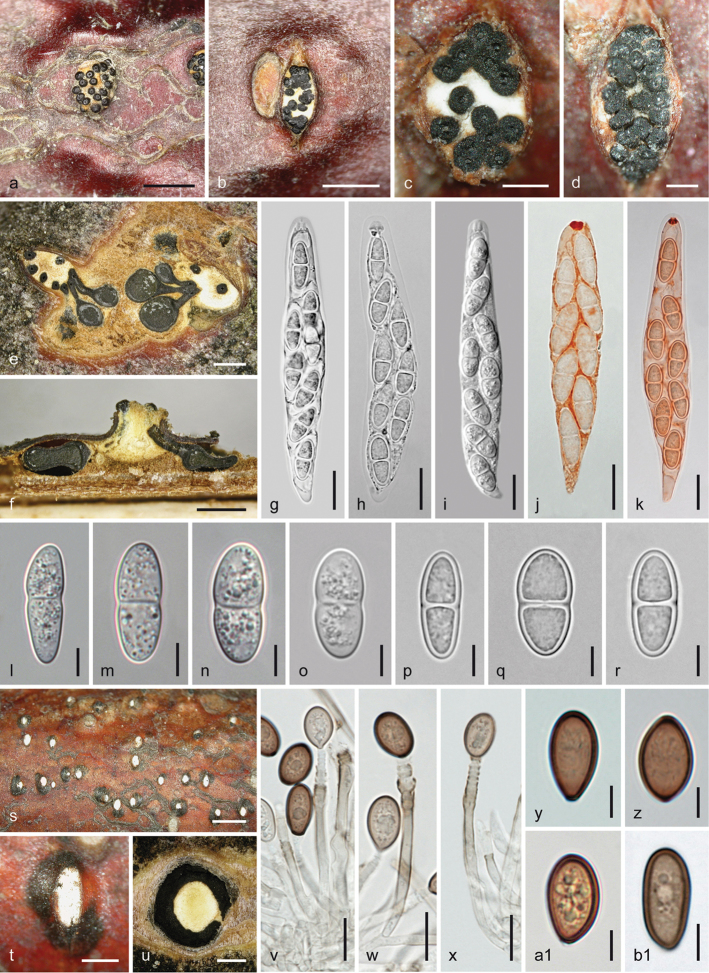
*Melanconis stilbostoma*. **a–r** Sexual morph **a–d** pseudostromata with ectostromatic discs in face view **e** cross section through 2 adjacent pseudostromata **f** vertical section showing 2 perithecia, ostiolar necks and central column **g–k** asci **l–r** ascospores **j, k** in aqueous Congo Red **s–b1** Asexual morph **s, t** conidiomata in face view **u** conidioma in cross section **v–x** conidiophores and conidiogenous cells **y–b1** α-conidia **v–b1** in 3% KOH **a, j, s, v–x** iso-epitype WU 31897 = W.J. 1543 **b–d**WU 31896 **e–g, i, k, o**WU 38241 **h, p, q**WU 36779 **l–n, a1** WU31899 **r**WU 37048 **t**WU 31894 **u**WU 15266 **y***M. betulinum* B700016529 **z***M. betulinum* B700016528 **a1** WU31899 **b1**WU 35970 = D143. Scale bars: 1 mm (**a, b**), 300 µm (**c, d**), 500 µm (**e, f, t, u**), 15 µm (**g–k**), 5 µm (**l–r, y–b1**), 2 mm (**s**), 10 µm (**v–x**).

##### Description.

***Sexual morph***: *Pseudostromata* 1.3–3.6(–4.5) mm diam., scattered to aggregated, slightly or distinctly projecting from bark surface, pulvinate with bluntly conical centre (projecting disc), circular to elliptical in outline; consisting of an ectostromatic disc and perithecia embedded in an entostroma around a central column and often chambers filled with conidia. *Ectostromatic discs* 0.4–2.4(–2.7) mm diam. or length, fusoid to circular, projecting from the bark surface to 0.5 mm, less commonly 1 mm including pseudostroma, white or yellow, brown when old, flat, concave or convex, often completely filled by tips of ostiolar necks; central column beneath disc brightly white to yellow, consisting of hyaline hyphae and colourless crystals. *Ostiolar necks* cylindrical, laterally or centrally attached on perithecia, convergent and densely and irregularly or evenly disposed in the disc or around the margin; visible part in the discs (106–)139–231(–283) µm (n = 68) diam., 1–25 per disc, shiny black, convex papillate, discoid with depressed centre or conical to cylindrical and projecting to 300 µm. *Entostroma* paler than surrounding inner bark, consisting of hyaline to white hyphae and bark cells, sometimes forming white patches. *Perithecia* (450–)540–700(–780) µm (n = 45) diam., arranged in valsoid configuration around and below central column, globose to subglobose, collapsing upon drying. *Peridium* pseudoparenchymatous, consisting of a dark brown small-celled outer and a hyaline to brownish, large-celled inner layer. *Hamathecium* absent at maturity. *Asci* floating free at maturity, (69–)80–123(–141) × (10–)13–18(–21) µm (n = 64), fusoid to oblong or narrowly clavate, with an apical ring distinct in water and staining in Congo Red but invisible or indistinct in 3% KOH, containing 4–8 biseriate or obliquely uniseriate ascospores. *Ascospores* (13.7–)16–19(–23) × (4.7–)6.5–8.5(–9.7) µm, l/w (1.9–)2.1–2.7(–3.6) (n = 186), first narrow, fusoid or oblong and with small roundish appendages (1.5–)2–5(–7.3) × (2.2–)3.3–5.5(–6.8) µm, l/w (0.3–)0.5–1.1(–1.7) (n = 60) within asci, later becoming broadly ellipsoid with rounded ends, symmetric or inequilateral, slightly constricted at the central to slightly eccentric septum, hyaline, thick-walled, smooth; appendages fugaceous and absent on released ascospores.

***Asexual morph*** acervular, intermingled with pseudostromata of the sexual morph or developing separately, conspicuous. First white tissue (central column) forming within the bark, becoming surrounded by sterile yellow margin and narrow discs rupturing bark epidermis, followed by the production of conidia in olivaceous to black chambers containing black conidial masses translucent though bark. *Conidiomata* 0.9–3.2 mm diam., subconical or pulvinate, more or less circular in outline, scattered or crowded.

*Covering discs* 0.3–1.2 mm long, slit-like to circular, flat to convex, shiny white to yellowish, becoming obscured by dark olivaceous brown to black conidial deposits forming patches to 2.7 mm diam., sometimes confluent to 1 cm; discs and pulvinate or conical columns beneath, consisting of dense *textura intricata* of hyaline hyphae and numerous colourless crystals, becoming brittle with age. *Conidiophores* emerging around the central column from a pseudoparenchymatous base, filiform, branched near the base and usually 1–3 fold asymmetrically at higher levels, first hyaline, turning brown from their tips; terminal *conidiogenous cells* (11.5–)18–33(–42.5) × (2–) 2.5–3.5(–4.5) µm (n = 47), more or less cylindrical, with up to 5 or 6 annellations, densely arranged, repetitive, producing α-conidia. *Conidia* (10.5–)12.5–15(–17.5) × (6.2–)7.2–8.5(–9.5) µm, l/w (1.3–)1.6–2(–2.7) (n = 260), oval, ellipsoid or subglobose, 1-celled, dark brown, thick-walled, smooth, with a few drops and a small scar. No β-conidia detected.

***Culture***: Colony on CMD at 16 °C forming irregular white and brown to ochre zones partly covered by aerial hyphae or hyaline, undifferentiated, forming brown spots and irregularly disposed conidiomata; on MEA at room temperature first white, later with broad white and brown zones with undulating margin and conidiomata forming mostly on the outer margin. Odour indistinct to fruity.

##### Distribution and ecology.

*Melanconis stilbostoma* occurs frequently on *Betula* spp. on the northern Hemisphere in Asia, Europe and North America (Barr 1978; Fan et al. 2016, 2018; Kobayashi 1970; Sogonov et al. 2008).

##### Other material examined.

(all on twigs of *Betula pendula* except where noted): **Austria**, Kärnten, Gallizien, near Wildensteiner Wasserfall, grid square 9453/3, 11 Jul 2007, W. Jaklitsch (WU 31896); St. Margareten im Rosental, village area, grid square 9452/4, 27 May 1992, W. Jaklitsch (WU 15266); Trieblach, below Cihuc, grid square 9452/2, 14 Apr 2001, W. Jaklitsch W.J. 1740 (WU 31895, BPI 872036; culture A.R. 3637); Wograda, grid square 9452/3, 27 May 1997, W. Jaklitsch W.J. 1080 (WU 31894); same area and host, 31 May 2000, W. Jaklitsch W.J. 1474 (BPI 871332); Zabrde, grid square 9452/4, 7 Aug 1993, W. Jaklitsch (WU 15191); Niederösterreich, Aspangberg-St. Peter, Außerneuwald, Höllergraben, grid square 8462/1, 24 May 2015, G. Koller (WU 36779); Edlitz, Königsberg, grid square 8562/2, 14 Jul 2007, W. Jaklitsch W.J. 3125 (specimen lost; culture MS = CBS 121894); Friedersbach, S and SO from the village, grid square 7457/2, 19 Aug 2001, W. Jaklitsch W.J. 1775 (BPI 872038; culture A.R. 3725); Neunkirchen, Gloggnitz, Saloder, village area, grid square 8361/2, 10 May 2015, G. Koller (WU 36752); Grimmenstein, between Eben and the Kulmriegel, grid square 8362/4, 14 May 2015, G. Koller (WU 36812); Thaures, grid square 7156/1, 21 Sep 1997, W. Jaklitsch W.J. 1109 (WU 37048); Weidlingbach, grid square 7763/1, 27 Jun 1999, W. Jaklitsch W.J. 1329 (WU 37049); Oberösterreich, Schärding, Raab, Rothmayrberg, Rothmayr, 10 Mar 2012, H. Voglmayr (WU 38241); St. Willibald, Großer Salletwald, at the road B 129 to Peuerbach, grid square 7648/1, 31 Dec 2011, H. Voglmayr (WU 31899); Vienna, Alsergrund, at the hospital AKH, grid square 7764/3, 23 Jul 1993, W. Jaklitsch (WU 15537); Favoriten, Rothneusiedl, grid square 7864/3, 4 Sep 1993, W. Jaklitsch (WU 15758); ibidem, 22 Jan 1994, W. Jaklitsch (WU 15559). **Czech Republic**, Bohemia, Malonty, Hodonický potok, grid square 7253/3, 25 Sep 2003, W. Jaklitsch W.J. 2427 (WU 31898). **Germany**, no collection data (type material B 700016528 and B 700016529 of *Melanconium betulinum* from B). **Italy**, Sicily, Etna, SW Linguaglossa, near I Due Monti, on *Betula aetnensis*, 18 Jun 2016, H. Voglmayr & W. Jaklitsch (WU 37047; culture D258). **Japan**, Nagano, Karuizawa, Mt. Asama, on Betula platyphylla Sukachev var. japonica (Miq.) Hara, 21 Sep 1965, T. Kobayashi (TFM FPH2710; culture MAFF 410225 = M3-9 = ME12). **Poland**, Narewka, NE Nowa Lewkowo, 27 Jul 2015, H. Voglmayr (WU 35970; culture D143).

##### Notes.

*Melanconis stilbostoma* and its basionym *Sphaeria stilbostoma* (α *papula*) were mentioned by Tulasne (1856), but the combination was invalid due to the lack of a generic diagnosis; it was, however, validated in Tulasne and Tulasne (1863). According to Ibai Olariaga, who examined the type in UPS, there are 3 scalps of *Betula* bark containing many clustered perithecia with black ostiolar necks erumpent through a white disc; neither asci nor spores were found, but brown α-conidia are present abundantly. As the type collection was distributed in Fries’ Scleromyceti Sueciae no. 145, we here lectotypify the species with the copy preserved in UPS, which we epitypify with a recent well-developed collection for which a culture and sequence data are available.

Several asexual morph names have been linked with *Melanconis stilbostoma*: *Melanconium bicolor* predates *Melanconis stilbostoma*, but there is no material extant in B, thus it cannot be checked; also *Quercus* but not *Betula* was given as host in the protologue. In addition, *Melanconis stilbostoma* is a well-known and well-defined name for the generic type of *Melanconis*. The second name is *Melanconium betulinum*, which is clearly a later synonym upon our examination of type material. *Melanconium elevatum* is another synonym. We have, however, not seen type material of this taxon, but the description and illustrations in Corda (1839) are conclusive. *Melanconis stilbostoma* is a very common fungus on birch throughout the northern hemisphere and likely the most conspicuous species of *Melanconis* due to the shiny white discs of both morphs, contrasting the dark conidial deposits. In older specimens, the latter may have olivaceous tones, but much less conspicuously than with *M. larissae*. The latter species differs also in a broad light zone present on its conidia. *Melanconis stilbostoma* was already cultured by Wehmeyer (1926b) on birch twigs from material, whose ascospore measurements were (13–)15–18 × 5–8 µm, corresponding to those of Barr (1978: 12–18.5 × 6.5–8(–9) µm). Wehmeyer (1941) gave (13–)15–19(–23) × (5–)6–7.5(–9) µm for ascospores, which is in accordance with our measurements ((13.7–)16–19(–23) × (4.7–)6.5–8.5(–9.7) µm); Kobayashi (1970) measured 13–25 × 4–7.5 µm, mostly 15–20 × 5–7 µm and Fan et al. (2016) gave (19–)21.5–23.5(–25) × (6–)7–8 μm, which is slightly larger. Wehmeyer (1941) noted for α-conidia from culture and exsiccata mostly 10–16 × 5.5–7.5 µm and 6.5–12 × 2–2.5 for β-conidia in culture; Barr (1978) found only α-conidia and measured 9–16.5 × 5–7.5 µm, which is in accordance with our observations from Europe (see above). Asian authors gave 9–16.5 × 5–7.5 µm (Kobayashi 1970) and (8.5–)9–14.5(–16) × (4.5–)5–6(–6.5) μm (Fan et al. 2016) for α-conidia, but, in some collections, they also found cylindrical to allantoid, unicellular, hyaline β-conidia, 9–11.5 × 1.5–2.5 µm (Kobayashi 1970) or (9–)10–11(–12.5) × (2–)2.5–3 μm (Fan et al. 2016).

### Validation of neotypification

Here we also validate the neotypification of *Melanconium pterocaryae*, the basionym of *Juglanconis pterocaryae* by Voglmayr et al. (2019), where the new requirement to explicitly state the MBT number in the typification proposal was missing:

#### Juglanconis pterocaryae

Taxon classificationFungiDiaporthalesMelanconidaceae

(Kuschke) Voglmayr & Jaklitsch, in Voglmayr, Castlebury & Jaklitsch, Persoonia 38: 150 (2017).

4E86330C-4989-579E-8C94-2AC3A6E4114E

≡ Melanconium pterocaryae Kuschke, Trudy Tiflissk. Bot. Sada 28: 25 (1913) (Basionym).

##### Typification.

Austria, Oberösterreich, Bad Hall, Kurpark, on corticated twigs of *Pterocarya fraxinifolia*, 20 Oct 2017, W. Jaklitsch (WU 39981, neotype of *Melanconium pterocaryae* here proposed; ex neotype culture D272 = CBS 144326; MBT 389379).

## Discussion

### Circumscription of the genus *Melanconis*, morphology and delimitation from morphologically similar genera

As already mentioned in the Introduction, the genus *Melanconis* historically has been considered a large, heterogeneous genus. Many species were removed to other genera in the past on morphological grounds or due to different associated asexual morphs: *Chapeckia* (Barr 1978), *Caudospora* (Starbäck 1889), *Hapalocystis* (Fuckel 1863), *Macrodiaporthe* (Petrak 1920), *Massariovalsa* (Saccardo 1882; Barr 1978), *Phaeodiaporthe* (Petrak 1920), *Pseudovalsa* (Ces and De Not 1863) and *Pseudovalsella* (Höhnel 1918). Only recently, species were relegated to other genera and families based on molecular phylogenetic analyses: *Alnecium* (Voglmayr and Jaklitsch 2014), *Caudospora* (Voglmayr and Mehrabi 2018), *Coryneum*/*Pseudovalsa* (De Silva et al. 2009), *Hapalocystis* (Jaklitsch and Voglmayr 2004), *Juglanconis* (Voglmayr et al. 2017, 2019), *Lamproconium* (Norphanphoun et al. 2016), *Melanconiella* (Voglmayr et al. 2012), *Phaeodiaporthe* (Voglmayr and Jaklitsch 2014), *Stilbospora*/*Prosthecium* (Voglmayr and Jaklitsch 2008, 2014).

All melanconis-like species form their fructifications in bark and lack black zones, which delimit the pseudostromata from surrounding bark tissue in genera like *Diaporthe*. The sexual morph in *Melanconis* sensu stricto is characterised by distinctly projecting white to yellowish ectostromatic discs, which continue as stromatic central columns downwards, by entostroma, which is optically scarcely different from internal bark tissue, by long cylindrical ostiolar necks, which converge in the disc, by hyaline bicellular ascospores with or without appendages, by absence of paraphyses at maturity and asci, which have an apical ring and are released from the subhymenium at maturity. Conidiomata of the asexual morph are acervular. They commonly produce two types of conidia, melanconium-like brown α-conidia and narrow hyaline to brownish β-conidia. Species of *Dendrostoma* in the Erythrogloeaceae (Jaklitsch and Voglmayr 2019; Jiang et al. 2019) also produce two types of conidia on the same conidiophores, but both are hyaline. Acervuli of *Melanconis*, however, particularly in *M. marginalis*, form chambers, in which first β-conidia are produced. Such chambers are still present when α-conidia are produced, but in the latest stages of maturation, the entire fertile region around the central column is filled with α-conidia and appears as a single locule. In species of the morphologically most similar genera *Melanconiella* (Voglmayr et al. 2012) and *Juglanconis* (Voglmayr et al. 2017, 2019), pseudostromata are less conspicuous and project to a lesser degree from the bark surface than in *Melanconis*. The central column in *Melanconiella* is usually grey, dull yellow to greenish, only rarely white and often poorly developed and ascospores may be hyaline or brown. The most striking difference between *Melanconis* and *Melanconiella* lies in the asexual morph. In *Melanconis*, each species produces α- and β-conidia in the same conidiomata, whereas each species of *Melanconiella* only produces a single type of conidia, either brown melanconium-like (corresponding to α-conidia) or hyaline discosporina-like conidia (corresponding to β-conidia). Species of *Juglanconis* only produce melanconium-like conidia, which have a gelatinous sheath (also present in a few *Melanconiella* spp.) and differ from the other genera by the presence of verrucae on the inner surface of the conidial wall.

### Molecular phylogeny, species numbers, concept and delimitation

In *Melanconiella*, 15 species have been recognised (Voglmayr et al. 2012; Fan et al. 2018) and five in *Juglanconis* (Voglmayr et al. 2017, 2019). Fan et al. (2016, 2018) included five species of *Melanconis* sensu stricto in their phylogenetic trees. Here we add three species, of which two are new. While all betulicolous species, except for the basal *M. betulae*, formed a highly supported clade, those on *Alnus* were scattered in between, so no general evolutionary pattern in host association could be revealed. Remarkably, within species, a commonly high genetic divergence and variability was observed (e.g. within *M. groenlandica*, *M. itoana*, *M. marginalis* and *M. stilbostoma*; see Fig. 1), contrary to *Melanconiella* and *Juglanconis*, where the species clades were genetically rather homogeneous (Voglmayr et al. 2012, 2017, 2019; Fan et al. 2018). This may, in part, be attributed to the wider geographic distribution and host range of these *Melanconis* species, but it may also indicate that they are within the process of evolutionary radiation and speciation. Although the species concept in *Melanconis* is primarily based on phylogenetic analyses, we consider morphological and ecological evidence as important criteria for taxonomic conclusions. The taxa on *Betula* spp. may be more or less easily distinguished by differences in the morphology of α-conidia and by ecology: α-conidia of *M. larissae* have a large light-coloured zone, those of *M. itoana* have a l/w ratio of > 3 and those of *M. betulae* and *M. groenlandica*, as given by the respective authors, are shorter than those of the other species, albeit similar. However, the latter two species occur on different host species: *M. betulae* on *Betula albosinensis*, *M. groenlandica* on *Betula maximowicziana*, *B. nana* and *B. papyrifera*.

Taxa on *Alnus* spp. may pose difficulties in differentiation. Ascospores of *M. alni* and *M. marginalis* differ in shape, size and particularly in appendages from each other. Nonetheless, all features are overlapping and, for example, ascospore appendages of *M. alni* are not always long and pointed, particularly in old fungarium specimens, but show some similarities with those of *M. marginalis*. In such cases, it is important to have the asexual morph in order to study its conidia, which are strikingly different from those of *M. marginalis*. The same applies to *Melanconis* accessions from the western North American *Alnus rubra*, where the co-occurring *M. pacifica* and *M. marginalis* can be reliably distinguished by their conidia (see, for example, also fig. 2 in Sieber et al. 1991).

The situation is particularly complex within *M. marginalis*, which splits up into four subclades in our phylogenetic analyses. Morphology amongst those subclades is very similar, measurements are heavily overlapping and only subtle differences or tendencies are recognisable. In addition to the lack of distinctive morphological characters, there is also a substantial amount of genetic variation within the two of the four subclades, for which several accessions are available, particularly within *M. marginalis* sensu stricto, which will certainly increase if more accessions from additional geographic areas and *Alnus* species and subspecies are added. Only a small part of the distribution area of *M. marginalis* is yet sampled. We, therefore, do not think that these subclades should be interpreted as different species, but as a single variable species. Acknowledging the geographical and genetic differentiaton, we decided to classify them as subspecies that may be within the process of speciation. Vicariant speciation may be the reason for splitting of the *M. marginalis* clade into two main clades, but the residual two clades that are only based on a single and two specimens, were gathered within a small restricted region in Austria and northern Italy. The internal structure of the whole clade may therefore change, in particular, if isolates from additional specimens collected in western and central Russia were added to the phylogenetic analyses and if sequences of all phylogenetic markers of Melanconis marginalis subsp. italica were included.

Misidentification of *M. alni* and *M. marginalis* is also prominent in GenBank sequences that were used in all published phylogenetic analyses including these species, resulting in an interchanged application of the names. Based on, as we now know, incorrect assumptions purported in the literature (e.g. Wehmeyer 1941) that *M. marginalis* is a North American and *M. alni* a European species, Central European accessions of *M. marginalis* were misidentified as *M. alni*. Vice versa, M.E. Barr misidentified her Canadian isolate from *Alnus rubra*, that is closely related to *M. alni* and here described as *M. pacifica*, as *M. marginalis*. Therefore, all sequences currently deposited in GenBank as *M. alni* actually represent *M. marginalis*, while those of *M. marginalis* belong to *M. pacifica*.

### Hosts

While *Juglanconis* is confined to the Juglandaceae, subtribus Juglandinae (Voglmayr et al. 2017, 2019), both *Melanconiella* and *Melanconis* occur on the Betulaceae. So far, species of *Melanconiella* primarily occur on the subfamily Coryloideae with the exception of *M. betulae* and *M. decorahensis*, which inhabit *Betula* (Voglmayr et al. 2012; Fan et al. 2018). In contrast, *Melanconis* is confined to *Alnus* and *Betula*, the sole genera of the subfamily Betuloideae. While all known *Melanconis* species are highly host specific on the generic level (i.e. no *Melanconis* species occurs on *Alnus* as well as *Betula* hosts), host specificity is less expressed and variable concerning their host species range. In addition, the same host species is commonly used by more than one *Melanconis* species. For instance, the widely distributed *M. stilbostoma* has been recorded from various species of *Betula*, which is likewise true for *M. groenlandica* (for confirmed hosts, see Table 1). Conversely, *M. betulae* is so far only known from a single host, *B. albosinensis*, which, however, is also host for *M. itoana* (Fan et al. 2016, 2018). For *Melanconis* species on *Alnus*, *M. alni* and *M. marginalis* show some host specificity but are not strictly host specific; while *A. glutinosa* and *A. alnobetula* are apparently only colonised by *M. alni* and *M. marginalis*, respectively, both species occur on *A. incana*. *Melanconis pacifica*, here described as a new species, seems to be host specific on *A. rubra*, which, however, also harbours *M. marginalis*. Therefore, the host species are of limited use for species identification and additional investigations are required to elucidate the host range of the various *Melanconis* species.

## Supplementary Material

XML Treatment for Melanconis

XML Treatment for Melanconis alni

XML Treatment for Melanconis betulae

XML Treatment for Melanconis groenlandica

XML Treatment for Melanconis itoana

XML Treatment for Melanconis larissae

XML Treatment for Melanconis marginalis

XML Treatment for Melanconis marginalis subsp. europaea

XML Treatment for Melanconis marginalis subsp. italica

XML Treatment for Melanconis marginalis subsp. marginalis

XML Treatment for Melanconis marginalis subsp. tirolensis

XML Treatment for Melanconis pacifica

XML Treatment for Melanconis stilbostoma

XML Treatment for Juglanconis pterocaryae
